# Skeletal pathologies in extant crocodilians as a window into the paleopathology of fossil archosaurs

**DOI:** 10.1002/ar.70079

**Published:** 2025-11-15

**Authors:** Alexis Cornille, Kyla Beguesse, Ewan Wolff, Clemens Zinner, Patrick Asbach, François Clarac, Florian Witzmann

**Affiliations:** ^1^ Dynamik der Natur, Leibnitz Institute for Research on Evolution and Biodiversity Museum für Naturkunde Berlin Germany; ^2^ Department of Biology Humboldt University Berlin Germany; ^3^ Comparative Zoological Osteological and Paleontological Pathology (CZOPP), LLC Durham North Carolina USA; ^4^ Biological Sciences Department North Carolina State University Raleigh North Carolina USA; ^5^ Paleontology research laboratory North Carolina Museum of Natural Sciences Raleigh North Carolina USA; ^6^ Honors College, University of New Mexico Albuquerque New Mexico USA; ^7^ Mountainside Animal Emergency & Speciality (MAES) North Vancouver British Columbia Canada; ^8^ Charité—Universitätsmedizin Berlin Department of Radiology Berlin Germany; ^9^ CR2P Sorbonne Université, Muséum National d'Histoire Naturelle, Centre National de la Recherche Scientifique, Centre de Recherche en Paléontologie—Paris (CR2P, UMR 7207) Paris France

**Keywords:** archosaur, bone, crocodilian, paleopathology, pathology

## Abstract

Crocodilians, together with birds, are the only extant relatives to many extinct archosaur groups, making them highly important for interpreting paleopathological conditions in a phylogenetic disease bracketing model. Despite this, comprehensive data on osteopathologies in crocodilians remain scarce. Here, we present the largest osteopathological survey to date of extant crocodilian skeletal material, comprising 927 individuals and 22,281 skeletal elements spanning all three extant crocodilian families. Our results reveal 1969 osteopathologies from 414 of all individuals (44.7%). Bone trauma is the most prevalent osteopathology, representing 43.9% of all pathologies observed, followed by inflammatory lesions (27.3%) and degenerative joint disorders (11.5%). Statistical analyses demonstrate significant correlations between adult body size, ontogeny, and trauma frequency, with large‐sized crocodilian species and older individuals sustaining substantially more injuries. The skull, particularly the dorsal cranial aspect, showed considerably higher involvement of trauma compared to the rest of the skeleton. We also show that non‐traumatic pathologies such as inflammation and degenerative joint disorders increase in larger crocodilian species and with ontogeny. These findings underscore the behavioral and physiological drivers of skeletal disease in modern crocodilians and offer key comparative data for interpreting osteopathologies in fossil archosaurs and archosauriforms.

## 1 INTRODUCTION

Extant crocodilians and birds represent the only surviving members of Archosauria, a clade that also includes non‐avian dinosaurs, pterosaurs, and the broader group of Crocodyliformes, which includes both modern crocodiles and their extinct relatives, a variety of Mesozoic groups that lie along the crocodilian lineage (Ezcurra & Butler, 2018; Nesbitt, 2011). Due to their phylogenetic position, crocodilians play a crucial role in research on the paleobiology of extinct archosaurs (Bell & Hendrickx, 2020; Brazaitis & Watanabe, 2011; Green et al., 2014; Isles, 2009; Kundanati et al., 2019; Seebacher et al., 1999; Wolff, 2007; Woodward & Farlow, 2023). While their general biology, physiology, and ecology offer valuable insights, it is also essential to consider the impact of diseases and how they influence their ecological interactions and evolutionary history.

The study of pathological conditions in extinct animals, that is, paleopathology, has proven to be a powerful tool not only for tracing the origins and evolution of diseases in deep time, but also for reconstructing aspects of the physiology and paleoecology of long‐extinct animals (Bishop et al., 2015; Ekhtiari et al., 2020; Fiorillo & Tykoski, 2023; Foth et al., 2015; Hanna, 2002; Haridy et al., 2019; Moodie, 1923; Pardo‐Pérez et al., 2020; Rothschild & Martin, 2006; Słowiak et al., 2021; Waldron, 2015; Witzmann et al., 2011). Paleopathology further allows the determination of the prevalence of pathologies in fossil assemblages, interpretation of their etiology, and comparison across assemblages or taxa (Baiano et al., 2024; Bell, 2010; Bennett, 2003; Brown et al., 2017; Martins et al., 2024; Pardo‐Pérez et al., 2019; Pardo‐Pérez, Kear, Gómez, et al., 2018a; Pardo‐Pérez, Kear, Mallison, et al., 2018b; Rothschild et al., 2003; Rothschild & Laub, 2013; Rothschild & Tanke, 2005; Tanke & Rothschild, 2014). Recent years have seen a marked increase in paleopathological research, particularly concerning Archosauriforms and archosaur taxa such as phytosaurs (Heckert et al., 2021; Witzmann et al., 2014), aetosaurs (Lucas, 2000), extinct crocodilians (Buffetaut, 1983; Katsura, 2004; Sawyer & Erickson, 1998), and, most extensively, dinosaurs (Anné et al., 2015, 2023; Baiano et al., 2024; Bertozzo et al., 2021, 2022; Brown et al., 2022; Hamm et al., 2020; Rothschild et al., 2003; Straight et al., 2009; Witzmann et al., 2008, 2011, 2016).

The diagnostic of a disease in the fossil record relies primarily on hard tissue preservation (i.e., bones and teeth). Accordingly, comparison with skeletal pathologies in extant vertebrates is indispensable as well as an extensive understanding of taphonomic artifacts (Assis et al., 2018; Bandyopadhyay et al., 2002; Biehler‐Gomez et al., 2021; Buikstra et al., 2017; Corron et al., 2017; Gummesson et al., 2017; Schotsmans et al., 2017). Thereby, two different approaches have been used in non‐mammalian paleopathology, which are not necessarily mutually exclusive (for a summary, see Witzmann & Asbach, 2022). The *trans‐phylogenetic* approach assumes only minor variations across phylogenetically disparate vertebrate clades in how skeletal diseases and injuries manifest, drawing on comparisons with human bone pathologies (Beatty & Rothschild, 2009; Ekhtiari et al., 2020; Rothschild, 2017; Rothschild et al., 2023; Rothschild & Martin, 2006). In contrast, *the phylogenetic approach* considers the differences in anatomy, physiology, and behavior across vertebrate clades and applies the extant phylogenetic bracket (EPB) (Bartosiewicz, 2021; Hamm et al., 2020; Witzmann et al., 2021; Wolff, 2007, 2008, 2009; Wolff et al., 2009). In this framework, an extinct taxon is bracketed by two closely related extant taxa. If a given character (here a disease) occurs in both living taxa, it is inferred that a homologous character was also present in the extinct taxon. For instance, paleopathology studies in dinosaurs are commonly bracketed between their two closest living relatives, crocodilians (the lower bracket) and birds (the upper bracket) (Hamm et al., 2020; Rega, 2012; Wolff, 2007, 2008, 2009; Wolff et al., 2009; Woodruff et al., 2022). While both approaches have merits, the phylogenetic method remains underused, partly due to the abundance of literature on human pathologies relative to that on non‐mammalian vertebrates. The authors posit that this disparity is driven by a number of factors including: a companion animal specific focus of veterinary orthopedic research; a lab animal specific focus of veterinary bone pathology research; a sampling bias in necropsy procedure for organs rather than bone(s); unless clinical history specifies an orthopedic‐related problem; and the absence of animal remains from most burial practices given cremation is more commonly elected. This imbalance limits our understanding of veterinary medicine dealing with osteopathology of reptiles, birds, amphibians, and fishes. Indeed, veterinary medicine references allocate only limited attention to skeletal diseases of reptiles and amphibians (Divers & Stahl, 2019; Garner & Jacobson, 2020; Huchzermeyer, 2003; Jacobson & Garner, 2020; Ladds, 2009). On the contrary, the paleopathology of dinosaurs and other fossil archosaurs benefits from a substantial body of literature on bone diseases in extant birds (Brothwell, 1993; Chinsamy & Tumarkin‐Deratzian, 2009; Dittmer et al., 2012; Echenique et al., 2019; Ehrlich et al., 2023; Espinosa et al., 2025; Fleming et al., 2004; Komosa et al., 2018; Rothschild & Panza, 2005; Rothschild & Rühli, 2007; Shivaprasad & Palmieri, 2012; Smits et al., 2005; Taivalkoski et al., 2021; Thorp, 1994; Tiemeier, 1941). In contrast, fewer studies address extant crocodilian bone pathology (Becker et al., 2018; Gorza et al., 2020; Huchzermeyer, 1986; Huchzermeyer et al., 2013; Kälin, 1936; Rothschild, 2010; Wolff, 2007, 2008). To date, only one study focused exclusively on the morphology and frequencies of skeletal pathologies in extant crocodilians: Kälin (1936) examined 40 complete skeletons in the Bavarian State Collection of Zoology (Munich; Germany) and documented bone pathologies in 10 individuals. Two other studies included extant crocodilians as part of a wider research scope: Wolff (2007) focused on extant and extinct archosaur bone pathologies of the oral cavity in which they examined 463 crocodilian individuals and reported 43 osteopathologies, while Rothschild (2010) examined non‐traumatic osseous pathologies in the postcranium of extant crocodilians and lizards and observed 20 pathologies among the 342 crocodilian specimens studied. Other reports of extant crocodilian bone pathologies are mere mentions in more general studies or isolated case reports; for example, Mook (1921) described a healed snout injury in *Crocodylus americanus* (i.e., *Crocodylus acutus*), Korschelt and Stock (1928) noted bite wounds on a caiman skull, Huchzermeyer et al. (2013) documented osteoarthropathy in several long bones of *Crocodylus niloticus*, Olatunji‐Akioye and Otuh (2015) reported metabolic bone disease (MBD) in a specimen of *Osteolaemus tetraspis*, Xu et al. (2020) described tail regeneration in an *Alligator mississippiensis*, Gorza et al. (2020) documented combined osteodystrophy, rickets, and bone marrow aplasia in a specimen of *Caiman latirostris*, Delene et al. (2020) discussed nutritional bone diseases and skin lesions in captive *C. niloticus*, Becker et al. (2018) conducted experimental research on osteometabolic diseases in *Caiman yacare*, and Steve Irvin documented the survival of a large *Crocodylus porosus* that lost a significant part of its lower jaw (Irwin, 1996). Even the standard work on pathologies of extant crocodilians, Huchzermeyer's (2003) “Crocodiles: Biology, Husbandry and Diseases,” devotes only a few pages to bone pathologies. Additionally, comprehensive large‐scale field studies have documented injuries and anomalies in living crocodilians. However, as these studies were based on live animals, most of the recorded pathologies were soft tissue related and do not reflect strict osteopathological data. Cott (1961) recorded 113 injured *C. niloticus* out of 548 in Uganda and Zimbabwe; Webb and Messel (1977) found injuries in 87 individuals and 12 with growth anomalies among 1345 *C. porosus* in Arnhem Land (Australia); Webb et al. (1983) recorded injuries in 449 individuals, and 31 with growth anomalies from 797 *Crocodylus johnstoni* in the Northern Territory (Australia); Gorzula (1978) found 33 injured individuals among 73 *Caiman crocodilus* in the Venezuelan Guayana; and Montague (1984) described injuries and abnormalities in 1073 *Crocodylus novaeguineae* in Papua New Guinea.

Thus, a comprehensive comparison of skeletal pathology in extant archosaurs has yet to be achieved, likely contributing to the continued reliance on human medicine in the paleopathological literature on fossil archosaurs.

The present study seeks to address this gap by providing a comprehensive overview of the morphology and frequency of osseous pathologies across all extant crocodilian taxa, based on firsthand examination of an extensive skeletal sample. Crocodilians represent ideal modern analogues for fossil archosaur paleobiology, not only due to their phylogenetic position as the lower bracket in the (EPB) for pterosaurs and dinosaurs, but also because aspects of their morphology and life history are broadly similar to basal archosaurs and archosauriforms like phytosaurs (Camp, 1930; Gregory, 1969; Gregory & Westphal, 1969; Jones & Butler, 2018; Lessner & Stocker, 2017; Stocker & Butler, 2013). As such, this study will contribute to our understanding of skeletal diseases in living crocodilians and provide a robust comparative framework for interpreting paleopathologies in fossil archosaurs.

Institutional abbreviations

**AMNH** American Museum of Natural History, New York City, USA

**BMNH** Natural History Museum, London, England

**FLMNH** Florida Museum of Natural History, Gainesville, USA

**FMNH** Field Museum, Chicago, USA

**MOR** Museum of the Rockies, Bozeman, USA

**NCSM** North Carolina Science Museum, North Carolina, USA

**PMJ** Phyletisches Museum Jena, Jena, Germany

**QM** Queensland Museum, Brisbane, Australia

**SMF** Senckenberg Museum, Frankfurt am Main, Germany

**SMNS** Staatliches Museum für Naturkunde Stuttgart, Stuttgart, Germany

**USNM** National Museum of Natural History, Washington DC, USA

**ZMB** Museum für Naturkunde, Berlin, Germany

**ZSM** Zoologische Staatssammlung, München, Germany.

## 2 MATERIALS AND METHODS

### 2.1 Sample

This study is based on a dataset comprising the skeletal remains of 927 extant crocodilian individuals, encompassing a total of 22,281 skeletal elements. These include 849 complete crania, 1583 mandibular rami, and 19,849 postcranial bones (17,822 postcranial bones + 2027 osteoderms) (Figure S1 in Supplementary Data). All three extant crocodilian families are represented: Crocodylidae (*n* = 476), Alligatoridae (*n* = 380), and Gavialidae (*n* = 61); an additional 10 individuals could not be assigned to the family level. Of the 927 individuals, our dataset is dominated by individuals with isolated skulls (*n* = 761; 82.1%), while 97 (10.5%) have both skull and postcranial elements, and 69 (7.4%) individuals present only postcranial elements.

**FIGURE 1:**
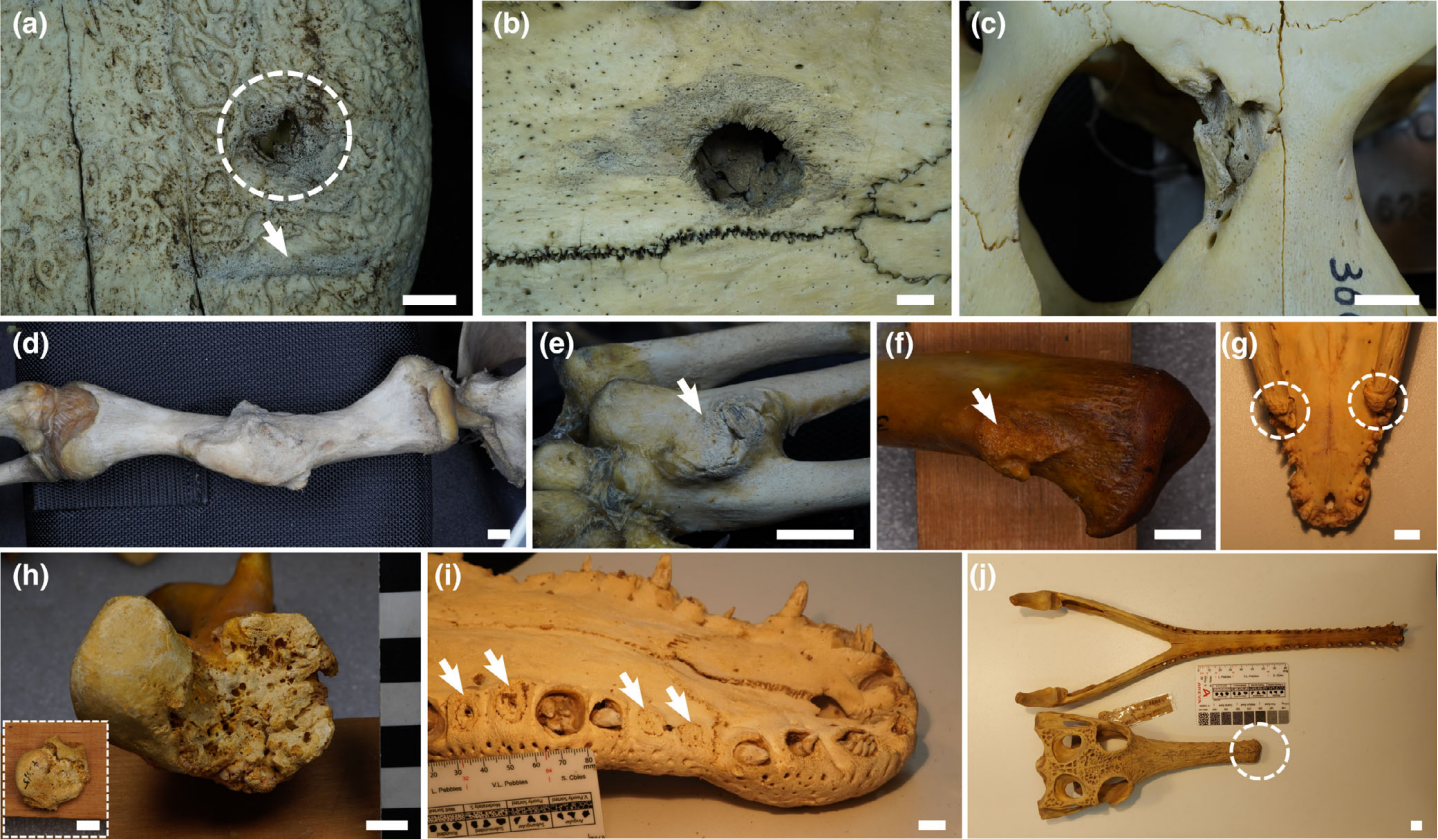
Bone trauma of extant crocodilians. (a) (UF 42872; *Alligator mississippiensis*) left maxillary dorsal aspect with puncture (dashed circle) and linear score (arrow); (b) (MNHN A‐5316; *Crocodylus porosus*) left maxillary ventral aspect with large circular bone surface perforation; (c) (ZMB 36628; *Crocodylus latirostris*) left palatine latero‐ventral aspect injury; (d) (MNHN 1873–538; *A. mississippiensis*) right humerus midshaft fracture callus; (e) (MNHN 1910–17; *A. mississippiensis*) metacarpals proximal aspect ankylosis (arrow); (f) (AMNH 31563; *A. mississippiensis*) tibia proximal aspect enthesophyte (arrow); (g) (SMF 28171; *Crocodylus niloticus*) bilateral mandibular amputation (dashed circles); (h) (AMNH 43315; *A. mississippiensis*) left humerus with latero‐distal articular surface broken loose (loose part in bottom left box); (i) (SMF 37173; *C. porosus*) left maxillary showing four sclerotic alveoli (arrows); (j) (SMF 28131; *Gavialis gangeticus*) snout amputation (dashed circle). All scale bars correspond to 1 cm.

### 2.2 Examination of skeletal elements

All skeletal elements were subjected to systematic macroscopic inspection to identify morphological and surface alterations. Observations were considered pathological if they occurred or developed during the animal's lifetime, evidenced by the presence of features that correlate with bone responses in well‐documented veterinary pathologic processes (Bigham‐Sadegh & Oryan, 2015; Craig et al., 2016; Divers & Stahl, 2019; Drumheller et al., 2023; Drumheller & Brochu, 2014; Garner & Jacobson, 2020; González, 2019; González‐Martín et al., 2022; Huchzermeyer et al., 2013; Huchzermeyer & Cooper, 2000; Jacobson & Garner, 2020; Olson & Carlson, 2017), thus excluding postmortem damages (e.g., breaks, preparation alterations, chemical alterations, scavenging traces…) (Corron et al., 2017). Each element was photographed regardless of its pathological status, with a metric scale for reference. Elements exhibiting potential pathology were further documented using overview and close‐up photographs taken with a Sony Alpha 6600 digital camera equipped with a FE 2.8/50 MACRO lens. These images served as the basis for subsequent diagnostic evaluation.

Thin‐sectioning was not permitted in most museum collections. However, selected specimens deemed likely to yield additional information were examined using computed tomography scanning with a Comet YXLON FF85 system at the Museum für Naturkunde (Berlin, Germany).

### 2.3 Diagnostic criteria

Given the fact that only some skeletal lesions are pathognomonic, even rigorous differential diagnoses often leave multiple plausible etiologies (Assis et al., 2018; Biehler‐Gomez et al., 2020; Hamm et al., 2020). Additionally, the high number of pathological individuals and pathologies observed prevented case‐by‐case diagnoses. Consequently, bone pathologies were classified into broad etiological categories to avoid overinterpretation (Baiano et al., 2024; Bell & Coria, 2013; Hanna, 2002).

Trauma (T): Diagnosed based on focal or multifocal, partial or complete discontinuity of the bone surface, accompanied by evidence of reactive bone formation indicating adjacent soft tissue or periosteal inflammatory response. Circular/sub‐circular or linear fashion lesions with well‐marginated edges in the presence of marginal reactive bone growth were considered strongly suggestive of bite marks. Traumatic fractures are based on evidence of partial or complete discontinuity of the bone surface with malalignment of the fracture site accompanied by expansive new bone formation (fracture callus) surrounding and stabilizing the fractured region, or nonunion (Craig et al., 2016; Drumheller & Brochu, 2014; Olson & Carlson, 2017) (Figure 1).

**FIGURE 2:**
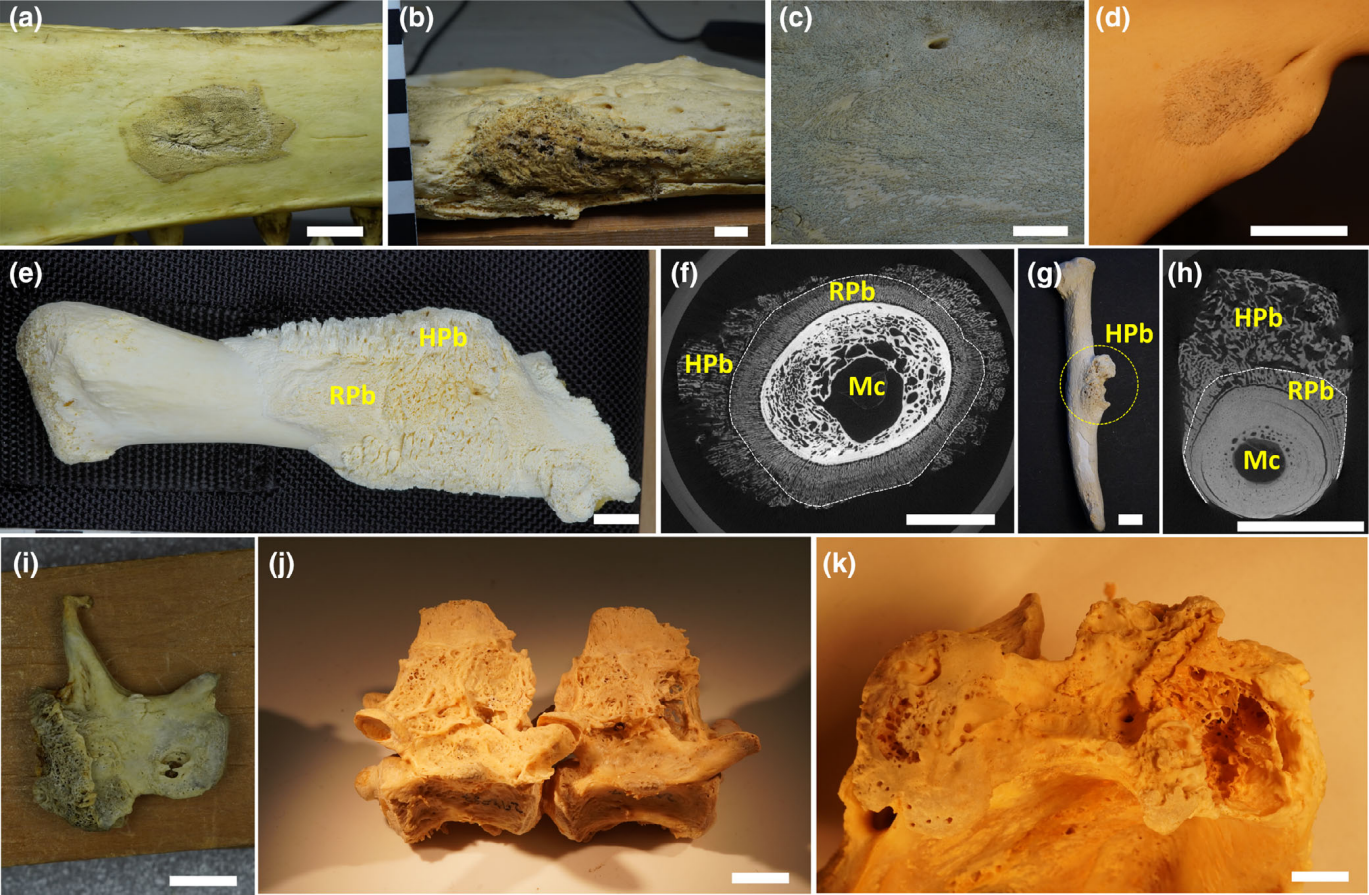
Bone inflammatory responses and infection of extant crocodilians. (a) (ZMB 36779; *Crocodylus acutus*) right splenial lesion with reactive bone formation; (b) (AMNH 31563; *Alligator mississippiensis*) mandible ventral aspect inflammation; (c) (ZMB 36674; *Crocodylus porosus*) oral cavity extensive thin layer of periosteal reactive bone formation on maxillary and premaxillary bones; (d) (SMF 55155; *Crocodylus intermedius*) right ectopterygoid inflammatory response with a focal thin layer of periosteal reactive bone; (e) (SMNS 106183; *C. acutus*) radius from an individual with systemic infection showing extensive periosteal reactive bone formation; (f) transverse microCT scan of radius (SMF 106183) highlighting an inner layer of radially oriented and outer layer of haphazardly oriented periosteal reactive bone with extensive cortical bone resorption; (g) (SMNS 6661; *A. mississippiensis*) fibula with large focal periosteal reactive bone from inflammatory reaction; (h) transverse microCT scan of fibula (SMNS 6661), highlighting the haphazardly oriented reactive bone; (i) (AMNH 97300; *Caiman yacare*) isolated caudal vertebra with osteolysis and reactive bone formation from an infectious process; (j) (USNM 2920178; *Alligator sinensis*) contiguous caudal vertebrae with extensive reactive bone formation from infectious process; (k) (USNM 211280; *C. acutus*) right quadrate articular surface showing aggressive infectious process. (HPb) haphazardly oriented pathological bone (RPb) radially oriented pathological bone; (Mc) medullary cavity. All scale bars correspond to 1 cm.

Inflammation (Im): Diagnosed on the presence of focal, multifocal, or diffuse bone surface resorption with accompanying reactive bone formation of indeterminate origin. Inflammatory reactions following trauma as a primary diagnosis were ignored based on the pathologic principles of inflammation developing as a response to trauma, and to avoid redundancy (Craig et al., 2016; Jacobson & Garner, 2020; Olson & Carlson, 2017) (Figure 2).

Infection (In): Diagnosed when lesions displayed aggressive and destructive bone resorption (osteolytic lesions) accompanied by reactive bone formation, occurring in focal, multifocal, or diffuse patterns. Widespread systemic infections were counted as a single pathology per individual. The authors acknowledge overlap in the manifestations of some inflammatory and infectious bone responses (Craig et al., 2016; Jacobson & Garner, 2020; Olson & Carlson, 2017) (Figure 2).

**FIGURE 3:**
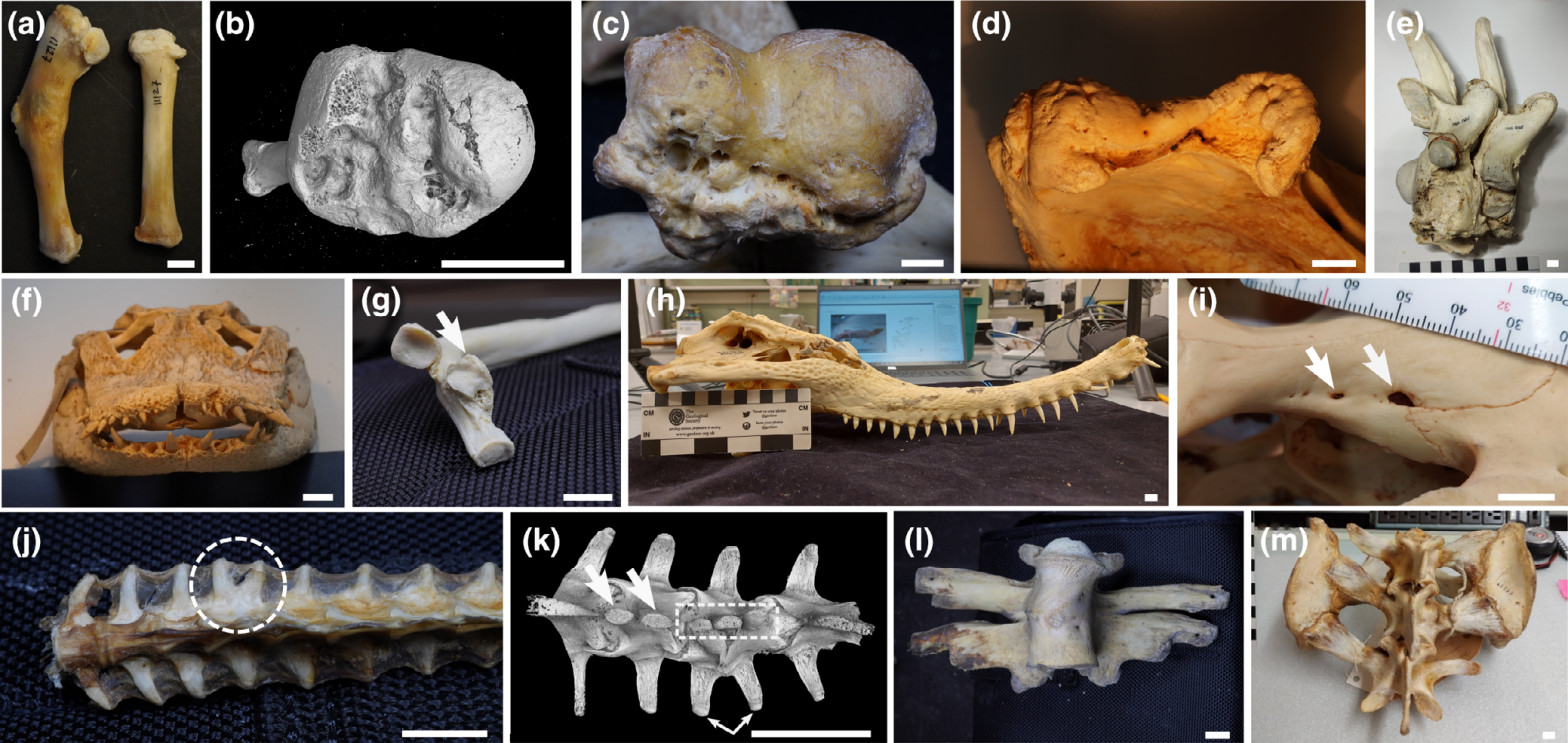
Bone degenerative joint disorders, developmental/congenital anomalies, and metabolic/nutritional disorders of extant crocodilians. (a) (SMNS 11127; *Crocodylus johnstoni*) right ulna and radius proximal articular surface with degenerative joint lesions likely resulting from the right ulna traumatic fracture; (b) 3D rendering from microCT scan of (SMNS 11127) ulna proximal articular surface in (a); (c) (MNHN 1963–22; *Crocodylus niloticus*) humerus distal articular surface degenerative joint disorder; (d) (SMF 28158; *C. niloticus*) right quadrate degenerative joint disorder; (e) (NHM 1969.1565; *C. niloticus*) cervical vertebrae ankylosis from degenerative joint disorder, and inflammation; (f) (SMF 55426; *A. mississippiensis*) lateral oblique orientation of teeth due to nutritional deficiencies associated with osteomalacia; (g) (SMNS 6663; *C. niloticus*) rib with supernumerary articular surface (arrow); (h) (FMNH 206755; *Tomistoma schlegelii*) snout upward bending anomaly; (i) (SMF 29079; *Caiman latirostris*) left palatine multiple foramen variation; (j) (AMNH 1887–910; *Alligator mississippiensis*) caudal vertebrae with duplicated transverse process (dashed circle); (k) 3D rendering from microCT scan of (AMNH 1887–910) with the duplicate transverse process (double arrows), triple neural spine (dashed rectangle) and duplicate on the adjacent vertebra (white arrows); (l) (MNHN 1944–230; *Crocodylus porosus*) segmentation failure of two thoracic vertebrae; (m) (USNM 66732; *C. porosus*) sacral anomaly with asymmetrical ribs and ilia articulations. All scale bars correspond to 1 cm.

Degenerative joint disorders (J): Diagnosed by alterations to the subchondral bone of articular surfaces, including marginal lipping, osteophyte formation, ankylosis, joint fusion, and subchondral bone surface collapse (Craig et al., 2016; Huchzermeyer et al., 2013; Olson & Carlson, 2017) (Figure 3).

Metabolic and/or nutritional disorders (M): Diagnosed on the basis of widespread, symmetrical alterations to bone morphology, such as increased cortical porosity, changes in apparent bone density, and shaft curvature. The authors recognized a reduced diagnostic precision due to limited access to internal structural information (Craig et al., 2016; Garner & Jacobson, 2020; Olson & Carlson, 2017) (Figure 3).

Developmental and/or congenital anomalies (D): Diagnosed where focal or symmetrical morphological bone anomalies could not be attributed to metabolic or nutritional etiology. Additional criteria include the presence of supernumerary bones, abnormal sutures, and atypical bone fusions not caused by inflammatory, infectious, or degenerative processes (Craig et al., 2016; Garner & Jacobson, 2020; Olson & Carlson, 2017) (Figure 3).

Neoplastic processes (N): Macroscopic recognition of bone neoplasia includes focal, multifocal, or regional, irregular “space‐occupying” lesions inside or in contact with the bone that cause bone destruction, new bone formation, and/or expansion/thickening of the bone. Malignant neoplasia exhibits rapid, aggressive growth that frequently invades and destroys the bone and/or metastasizes to other soft tissues/organs in the body, whereas benign neoplasms are usually slow growing, do not invade the bone, spread to the surrounding soft tissues, or metastasize throughout the body. The authors recognized the difficulty in assessing a definitive diagnosis in the absence of radiological, histological, and adjacent soft tissue analyses (Craig et al., 2016; Garner & Jacobson, 2020; Olson & Carlson, 2017).

Undetermined (Idiopathic) pathologies (U): This category includes all pathological alterations that could not be confidently assigned to any of the above (Figure S3 supplementary data).

Individual variations (V): Mild morphological anomalies deemed non‐pathological were noted for reference but excluded from pathology counts.

All pathologies were recorded in a dedicated table. Each individual exhibiting bone lesions was assigned counts for each relevant etiological category (T, Im, In, J, M, D, U, V), corresponding to the number of observed instances of each pathology type.

### 2.4 Bone trauma as a proxy to behavior in extant crocodilians

Bone trauma provides the most direct evidence for behavioral interpretation, as it results from interactions with conspecifics, other animals, or the environment. We therefore assessed the relationship between bone trauma and various biological and morphological categories:

#### 2.4.1 Size categories

To evaluate the effect of crocodilian adult body size on trauma frequency, individuals were grouped into three major size categories based on the species' maximum total body length (TL) at maturity (Grigg & Kirshner, 2015). Individuals exhibiting exceptionally large or small sizes outside the expected range were still assigned to their species size category.

Small‐sized species (TL< 2.5 m): *Alligator sinensis*, *C. crocodilus*, *C. latirostris*, *C. yacare*, *O. tetraspis*, *Paleosuchus palpebrosus*, *Paleosuchus trigonatus*.

Medium‐sized species (2.5≤ TL≤ 4 m): *C. johnstoni*, *Crocodylus moreletii*, *C. novaeguineae*, *Crocodylus rhombifer*, *Crocodylus siamensis*, *Mecistops cataphractus*.

Large‐sized species (TL >4 m): *A. mississippiensis*, *C. acutus*, *Crocodylus intermedius*, *C. niloticus*, *Crocodylus palustris*, *Crocodylus porosus*, *C. porosus* X *C. siamensis* hybrids, *Gavialis gangeticus*, *Melanosuchus niger*, *Tomistoma schlegelii*.

Our sample comprises 590 large‐sized individuals (63.6%), 88 medium‐sized individuals (9.5%), 238 small‐sized individuals (25.7%), and 11 individuals of undetermined size (1.2%), due to a lack of species‐level identification.

#### 2.4.2 Snout shapes

Bone trauma frequencies were analyzed across three broadly accepted snout shapes: brevirostrine, mesorostrine, and longirostrine.

Brevirostrine (broad, U‐shaped snout): *A. mississippiensis*, *A. sinensis*, *C. latirostris*, *C. moreletii*, *C. palustris*, *C. yacare*, *M. niger*, *O. tetraspis*, *P. palpebrosus*, *P. trigonatus*.

Mesorostrine (moderately elongated, V‐shaped snout): *C. acutus*, *C. crocodilus*, *C. intermedius*, *C. niloticus*, *C. novaeguineae*, *C. porosus*, *C. porosus* X *C. siamensis*, *C. rhombifer*, *C. siamensis*.

Longirostrine (elongated, mediolaterally narrow snout): *C. johnstoni*, *G. gangeticus*, *M. cataphractus*, *T. schlegelii*.

Snout shapes were assigned following Grigg & Kirshner (2015), except for *C. siamensis*, which we categorized as mesorostrine based on its strong morphological resemblance to *C. porosus* (Drumheller & Wilberg, 2020). The dataset includes 339 brevirostrine individuals (36.6%), 475 mesorostrine individuals (51.2%), 103 longirostrine individuals (11.1%), and 10 individuals (1.1%) with an indeterminate snout shape, due to a lack of species‐level identification.

#### 2.4.3 Total body length

Total body length (TL) was recorded from the specimen tag and/or provided documentation. If absent, size estimation from head width, length, or femoral length was used. Measurements were taken directly on‐site using a ruler or digitally from photographs using the software ImageJ 1.53 t. Body size estimation based on head width has proven to be very conservative and reliable across all extant crocodilian taxa regardless of the snout shape and was preferred when available (O'Brien et al., 2019). Head width (HW) was measured across the lateral margins of the quadrate bones and converted to total body length (TL) using the regression equation: (ln(TL) = 0.8024*(ln(HW)) + 3.05) (O'Brien et al., 2019). When head width was unmeasurable, head length was used and converted to total body length using the standard head‐to‐body ratio of 1:7 (Fukuda et al., 2013). If the cranium was absent, femur length was used following the equation (TL) = 16.45 + 14.45*(femur length) as in Farlow et al. (2005). Though such estimations may result in over‐ or underestimates, they serve our purpose of establishing an ontogenetic sequence for comparative analysis of osteopathological frequencies within size categories (i.e., large, medium, and small). Individuals with indeterminable (TL) were excluded from size‐related pathology analyses. Total body length in our sample ranges from 29.20 cm (*M. niger*, SMF 30103) to 553.64 cm (*C. acutus*, AMNH 7139). Maturity stages were not considered due to the inherent uncertainty in estimating maturity from size alone, given intraspecific, geographic, and sexual variability (Grigg & Kirshner, 2015).

#### 2.4.4 Sex

Information about sex determination was retrieved from information on the specimen tag and/or provided documentation. Bone trauma frequencies were analyzed in relation to sex (male, female, or unknown). Sex determination from skeletal remains is generally not achievable in crocodilians, except in *G. gangeticus*, where the sexually dimorphic ghara leaves diagnostic traces on the bone in the narial region (Hone et al., 2020). The sample comprises 35 females (3.8%), 48 males (5.2%), and 844 individuals of unknown sex (91%).

#### 2.4.5 Origin

Information about the origin was retrieved from information on the specimen tag and/or provided documentation. Bone trauma was also evaluated against origin (captivity, wild, or unknown). Regarding origin, 75 individuals were from captivity (8.1%), 501 from the wild (54%), and 351 from unknown provenance (37.9%). The authors recognized the importance of the differentiation between captive and wild animals in pathological studies; as such, comparison was considered and indicated in some analyses. Nonetheless, the aim of the present study is to provide an extensive presentation of broad osteopathological categories in extant crocodilians; thus, the small number of captive animals is here considered to have slight consequences. Additionally, it is fair to assume that the crocodilian skeletal remains of unknown origin in our sample were primarily wild caught from the late 19th and the early 20th century during the colonial period, when a boom in the global trade of natural history museum collections happened (Coote et al., 2017).

#### 2.4.6 Regional bone trauma and non‐traumatic osteopathology

Distribution of osteopathologies across the skeleton was compiled using a supplementary dataset where pathologies that affect multiple bones, such as some traumas, systemic infections, generalized metabolic and nutritional bone diseases, or degenerative joint disorders, were recorded for each bone affected. The new resulting dataset comprises 2077 osteopathological observations.

Trauma frequencies were recorded across different anatomical regions, including: cranium, mandibles, dentition, oral cavity, osteoderms, cervical vertebrae and ribs, thoracic vertebrae and ribs, gastralia, interclavicle, pectoral and pelvic girdles, forelimbs (humeri, radii/ulnae, manus), hindlimbs (femora, tibiae/fibulae, pes), sacral vertebrae, caudal vertebrae, and hemal arches. These data were used to generate a heatmap depicting the anatomical distribution of traumas, expressed as the relative frequency of trauma per region.

The non‐traumatic osteopathologies were categorized into three main anatomical regions: the skull, the axial postcranial skeleton, and the appendicular skeleton.

#### 2.4.7 Statistical analyses

Our pathological dataset is characterized by potential zero‐inflated count data, overdispersion, non‐normal distribution, and unequal sample sizes. Therefore, we opted for a negative binomial statistical model for all statistical analyses. Comparison of models fit (AIC) showed better results for the negative binomial model over a zero‐inflated negative binomial model in all analyses. Post‐hoc pairwise comparison and Tukey adjustment using estimated marginal means were used systematically (Supplementary Data S2).

Total body length (TL) was log‐transformed prior to analyses to improve visual clarity, interpretability (fold‐changes), better model fit for count data, and meet the assumptions of generalized linear modeling. Also, negative binomial models incorporating log (TL) consistently yielded lower AIC values and reduced residual deviance relative to models using untransformed TL, indicating superior model fit (e.g., trauma/TL = 1394 (AIC); trauma/LogTL = 1382 (AIC)). Additionally, we tested the normal distribution of total body length (TL) across the three different size categories using a Shapiro–Wilk test and density plots (Supplementary Data S2).

Finally, statistical results with *p*‐values equal to or lower than 0.05 were considered significant. All statistical tests were carried out in the software R 4.3.2 and RStudio 2023.12.0 (R Core Team, 2024).

## 3 RESULTS

### 3.1 General crocodilian osteopathology

**FIGURE 4:**
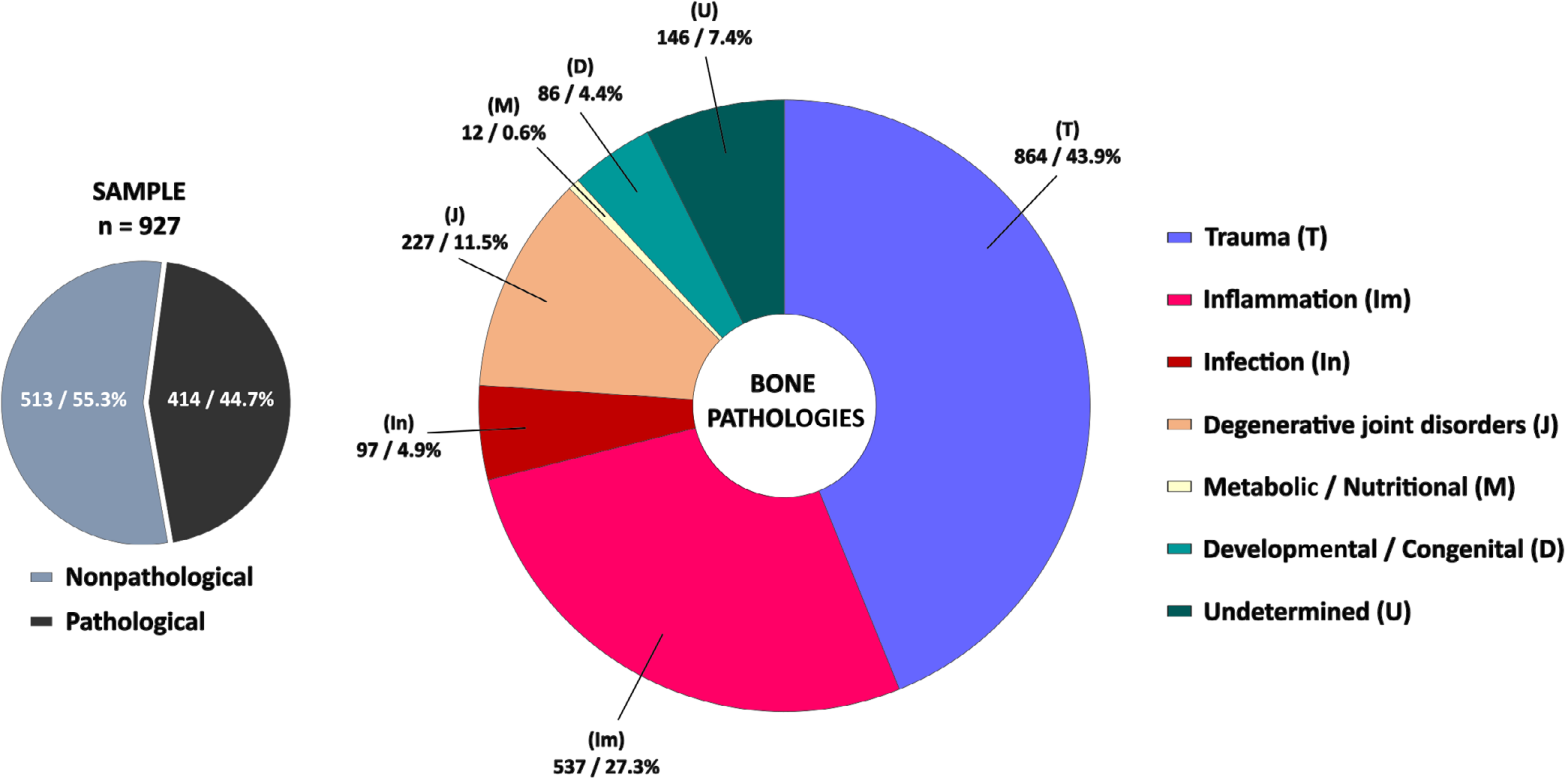
General frequencies of osteopathology categories in extant crocodilians.

Bone pathologies were observed in 414 out of 927 individuals (44.6%). A total of 1969 bone lesions were recognized on the 22,281 skeletal elements checked, from which trauma is the most observed type of pathology, with 864 lesions reported (43.9%). The second most observed pathology is inflammation, with 537 lesions (27.3%). Less commonly observed are degenerative joint disorders, with 227 diagnoses (11.5%), pathologies of undetermined etiology, attributed to 146 observations (7.4%), infection, represented by 97 lesions (4.9%), and developmental and congenital anomalies, with 87 observations (4.4%). Metabolic and nutritional disorders are rare, with 12 observations (0.6%), and no definitive neoplastic lesions were recognized based on our diagnostic criteria, and tentative bone neoplasia were assigned under the undetermined etiology category (Figure 4 and Tables 1 and 2 in supplementary data S2).

### 3.2 Bone trauma

#### 3.2.1 Size categories and trauma

Individuals of undetermined size were excluded from analyses examining the relationship between size categories and trauma (*n* = 11; 1.2% of the total sample). Thus, the analyzed dataset includes 916 individuals across small, medium, and large size categories, accumulating 852 traumas.

**FIGURE 5:**
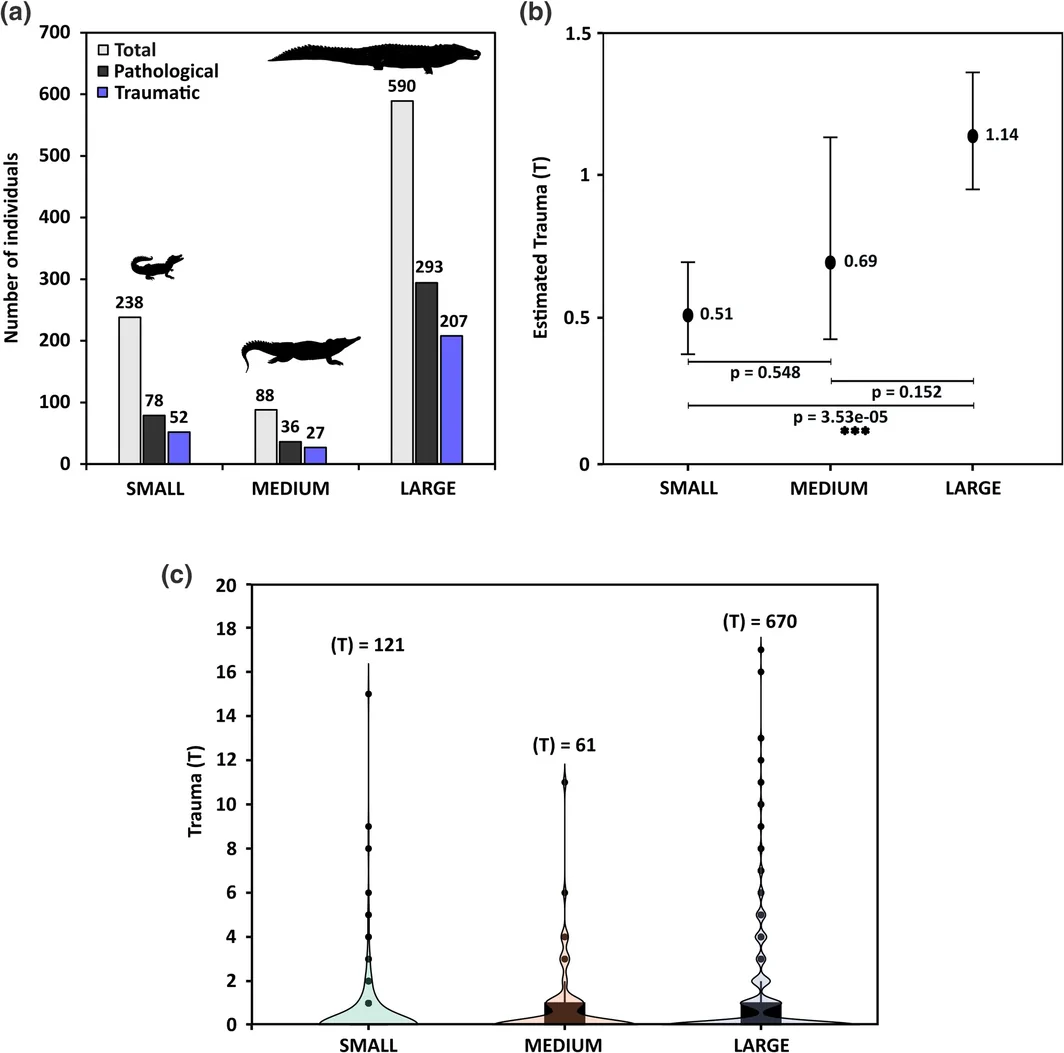
Bone trauma frequencies in large‐, medium‐, and small‐sized extant crocodilian species. (a) Total sample size, pathological individuals, and traumatic individuals; (b) estimated means of bone trauma with 95% CI and pairwise comparison *p*‐values (*** highly significant); (c) violin plot and box plot of bone trauma count distribution.

Large‐sized individuals represent the most abundant group with 590 individuals (64.4% of the total size category sample), with 293 identified as pathological (49.7% of the large‐sized sample), and 207 exhibiting traumas (35.1% of the large‐sized sample). Small‐sized individuals constitute the second largest group (*n* = 238; 26%), including 78 pathological individuals (32.8% of the small‐sized sample), and 52 traumatic individuals (21.8% of the small‐sized sample). Medium‐sized individuals are the least represented (*n* = 88; 9.6%), with 36 pathological individuals (40.9% of the medium‐sized sample), and 27 traumatic individuals (30.7% of the medium‐sized sample) (Figure 5a).

Figure 5c illustrates the distribution of trauma counts across size categories. Small‐sized individuals predominantly exhibit 0 or 1 trauma, with a few outliers and a maximum of 15 (total (T) = 121; 14.2% of 852). Medium‐sized individuals display a slightly broader distribution, with most trauma counts between 0 and 3 and a range up to 11 (total (T) = 61; 7.2% of 852). Large‐sized individuals show the widest non‐zero distribution up to a maximum of 17 (total (T) = 670; 78.6% of 852). All three size categories exhibit zero‐inflated distributions with medians of zero (Figure 5c). Consequently, we used a negative binomial statistical model to compare traumatic injury frequencies among size categories. Large‐sized individuals show the highest proportion of bone trauma with a mean of 1.14 (T) (95% CI: 0.949–1.359), followed by medium‐sized individuals with 0.69 (T) (95% CI: 0.425–1.131), and small‐sized individuals with 0.51 (T) (95% CI: 0.372–0.694). A post‐hoc pairwise comparison and Tukey adjustment returned no significant differences in trauma between small‐ and medium‐sized individuals (overlapping 95% CI, mean ratio = 0.733, *p*‐value = 0.548), and between medium‐ and large‐sized individuals (overlapping 95% CI, mean ratio = 0.61, *p*‐value = 0.152). However, the comparison between small‐ and large‐sized individuals shows a significant difference (no overlap in the 95% CI, mean ratio = 0.448, *p*‐value = 3.53e‐05) (Figure 5b). These results indicate that large‐sized individuals experience about twice as much bone trauma as small‐sized crocodilians (2.23‐fold increase) (Table 2 in supplementary data S2).

#### 3.2.2 Snout shape

In the same manner as for individuals of undetermined size category, individuals of undetermined snout shape were excluded from analyses (*n* = 10; 1.1% of the total sample). Accordingly, the analyzed dataset includes 917 individuals across mesorostrine, brevirostrine, and longirostrine, accumulating 852 traumas.

Mesorostrine crocodilians are the most represented snout shape category with 475 individuals (51.8% of the total snout shape sample), from which 218 individuals are considered pathological (45.9% of the mesorostrine sample), and 153 individuals show bone trauma (32.2% of the mesorostrine sample). The second most abundant category is the brevirostrine crocodilians with a sample of 339 individuals in total (37% of the total snout shape sample), 156 pathological individuals (46% of the brevirostrine sample), and 111 individuals that bear bone trauma (32.7% of the brevirostrine sample). The least represented snout shape category is the longirostrine crocodilians with 103 individuals in total (11.2% of the total snout shape sample), 33 pathological individuals (32% of the longirostrine sample) and only 22 individuals showing bone trauma (21.4% of the longirostrine sample). A negative binomial model was fitted to assess differences in trauma frequency among snout shape categories. Estimated marginal means indicated that brevirostrine taxa had the highest mean trauma count (total (T) = 337; mean (T) = 0.99; 95% CI: 0.78–1.27), followed closely by mesorostrine taxa (total (T) = 456; mean (T) = 0.96; 95% CI: 0.78–1.18). In contrast, longirostrine taxa exhibited a lower estimated trauma count (total (T) = 59; mean (T) = 0.57; 95% CI: 0.36–0.92). The confidence interval of longirostrine individuals is marginally lower than mesorostrine and brevirostrine individuals, and raw statistical comparison shows a significant difference in mean trauma count between longirostrine and brevirostrine individuals (raw *p*‐value = 0.0425). Nonetheless, after post‐hoc pairwise comparison and Tukey adjustment, we find no significant differences: brevirostrine against longirostrine individuals (*p*‐value = 0.105), brevirostrine against mesorostrine individuals (*p*‐value = 0.975), and longirostrine against mesorostrine individuals (*p*‐value = 0.123) (Table 2 in supplementary data S2).

#### 3.2.3 Sex

Negative binomial model statistical analyses reveal that the estimated marginal mean trauma count is higher in males (mean (T) = 0.98; 95% CI: 0.56–1.72) than in female individuals (mean T = 0.54; 95% CI: 0.26–1.12). However, this difference is not statistically significant (*p*‐value = 0.207) (Table 2 in supplementary data S2).

#### 3.2.4 Origin

Estimated marginal means indicate that captive individuals have a higher trauma frequency (mean (T) = 1.69; 95% CI: 1.02–2.82) than wild specimens (mean (T) = 0.82; 95% CI: 0.67–1.01). The non‐overlapping confidence intervals and a negative binomial model statistical analysis show a significant difference in trauma count within origin, with captive individuals exhibiting about twice as much trauma as wild individuals (*p*‐value = 0.0103) (Table 2 in supplementary data S2).

#### 3.2.5 Ontogeny

**FIGURE 6:**
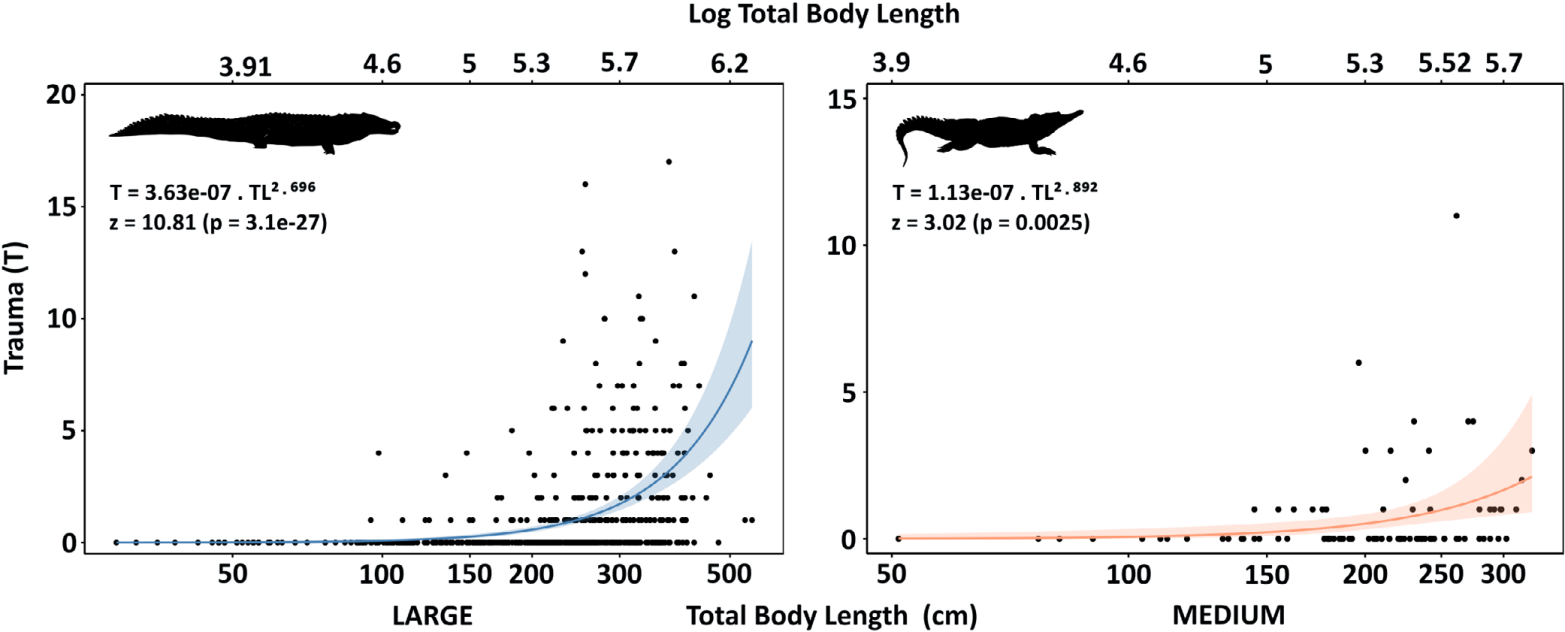
Bone trauma counts against the total body length in large‐ and medium‐sized extant crocodilians, with regression lines and equations, 95% confidence intervals (shaded areas), Z statistics and corresponding *p*‐values.

To visualize the effect of growth on the incidence of bone trauma, we plotted the counts of trauma against the estimated total body length of our crocodilian sample within each size category (i.e., large, medium, and small) to prevent overlapping of different ontogenetic stages. A Shapiro–Wilk test and density plots show that total body length (TL) is normally distributed in small‐sized individuals (*p*‐value = 0.39) and medium‐sized individuals (*p*‐value = 0.46), but shows a slight right skew in large‐sized individuals (*p*‐value = 0.003) (Figure S2 in supplementary data). A negative binomial regression trend line was calculated and fitted to the models with Log(TL) against (T). Results show a significant positive association between Log (TL) and frequencies of trauma (T) in large‐sized individuals (*z* = 10.81; *p*‐value = 3.1e‐27) and medium‐sized individuals (*z* = 3.02; *p*‐value = 0.0025) (Figure 6). In both size categories, growth is correlated with an increase in the number of bone traumas observed, with the strongest evidence in large‐sized species.

The negative binomial regression line model did not converge for the sample of small‐sized individuals, and no statistical support for a relationship between total body length and trauma in small‐sized crocodilian species was found (see supplementary data S2).

#### 3.2.6 Skeletal heatmap of trauma

**FIGURE 7:**
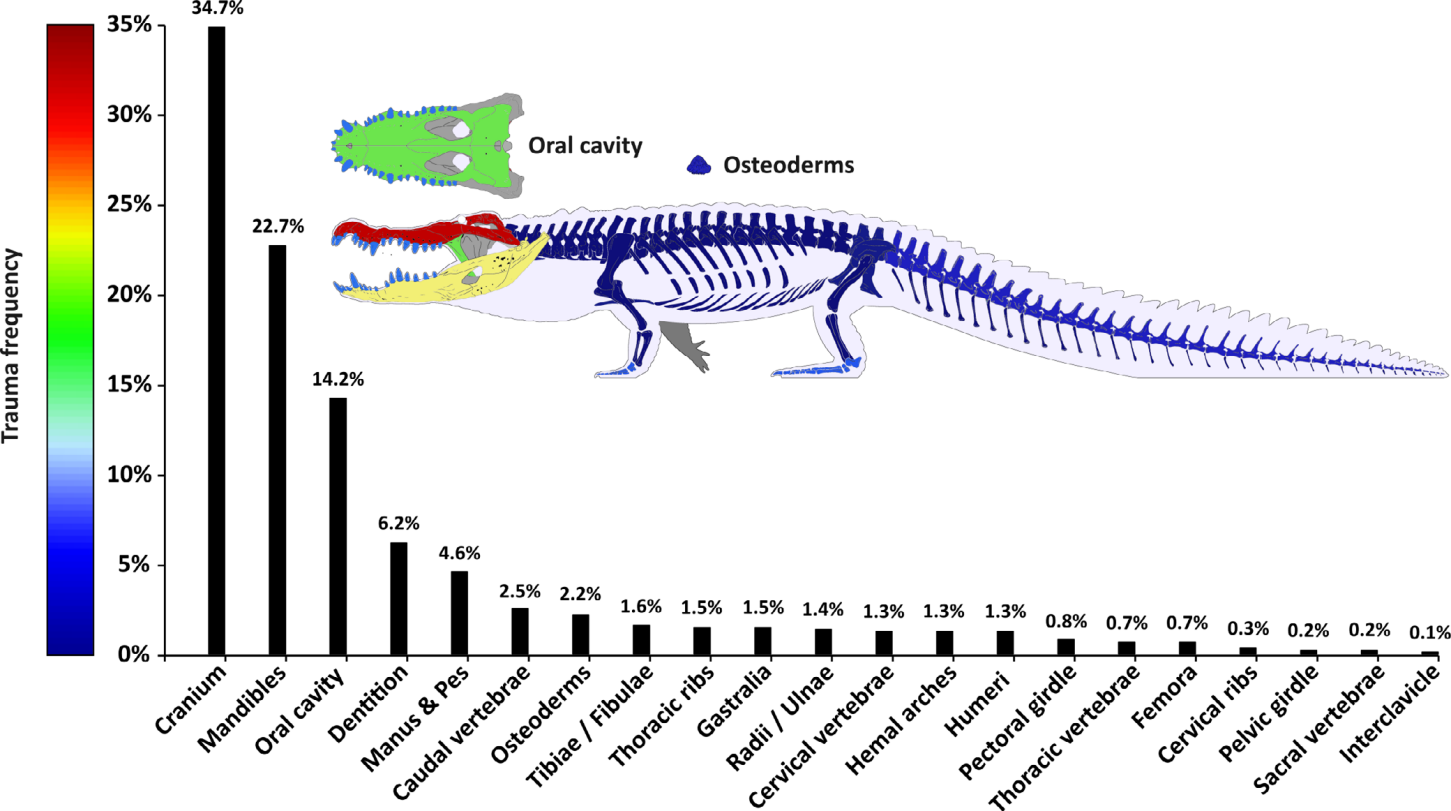
Heatmap of bone trauma frequencies by anatomical regions in extant crocodilians. Drawing of *Crocodylus siamensis* skeleton used with permission of Lamborlobator (https://www.deviantart.com/lamborlobator).

A heatmap of the anatomical distribution of trauma reveals a strong offset between the skull region and the postcranial skeleton (Figure 7). The skull bears 79.9% (total (T) = 680) of the 873 bone traumas observed in total. Distribution shows that the dorsal aspect of the cranium is the most impacted (total (T) = 304; 34.8%), followed by the mandibles (total (T) = 198; 22.7%), the oral cavity (total (T) = 92; 14.2%), and the dentition (total (T) = 54; 6.2%). In the postcranial region, manus and pes bones have the highest trauma frequency with 4.6% (total (*T*) = 40); the rest of the postcranial skeleton shows very low trauma frequencies with results equal to or below 2.5%.

#### 3.2.7 Estimated mean trauma by taxa

A calculation of the estimated mean trauma counts for each taxon was compiled (Table 3 in supplementary data S2). Results show that the wide confidence intervals (95% CI) and small sample sizes of some taxa limit interpretability and comparability. Therefore, we do not discuss those results.

### 3.3 Non‐traumatic osteopathologies

#### 3.3.1 Skeletal distribution of non‐traumatic osteopathologies

The skull bears 762 non‐traumatic bone pathologies. Within the skull region, bone inflammation is the most frequent pathology (total (Im) = 382; 50.1%), followed by pathologies of undetermined etiology (total (U) = 114; 15%), degenerative joint disorders (total (J) = 106; 13.9%), and infections (total (In) = 88; 11.5%). Developmental and congenital anomalies account for 7.7% (total (D) = 59), and metabolic and nutritional disorders (M) are rare, with only 13 observations (1.7%) (Table 4 in supplementary data S2).

In the postcranial axial skeleton, 357 non‐traumatic bone pathologies were observed. Within the postcranial axial region, infectious lesions are the most common observations (total (In) = 127; 35.6%), followed by degenerative joint disorders (total (J) = 106; 29.7%), inflammation (total (Im) = 105; 29.4%), and developmental and congenital anomalies represent 4.5% (total (D) = 16) of observed pathologies, while pathologies of undetermined etiology represent 0.8% (total (U) = 3) (Table 4 in supplementary data S2).

In the appendicular skeleton, non‐traumatic bone pathologies represent 432 observations. Within the appendicular region, bone infection is again dominant (total (In) = 198; 45.8%), inflammation represents 28% (total (Im) = 121), degenerative joint disorders accumulate to 16% (total (J) = 69), pathologies of undetermined etiology to 6.9% (total (U) = 30), and developmental and congenital anomalies to 3.2% (total (D) = 14) (Table 4 in supplementary data S2).

### 3.4 Bone inflammation and degenerative joint disorders

A comparison of degenerative joint disorder (J) counts between wild and captive individuals gives an expected mean (J) of 0.52 in captive crocodilians and 0.24 (J) in wild crocodilians, with a rate ratio of 0.47, which indicates that wild individuals have, on average, 53% less degenerative joint disorders than captive individuals in our dataset. Nonetheless, a negative binomial statistical comparison showed no significant effect on origin (*p*‐value = 0.139). Therefore, we do not apply a distinction between individuals from captivity and from the wild in the next sections.

#### 3.4.1 Size categories

Small‐sized individuals exhibit a mean bone inflammation (Im) frequency of 0.30 (95% CI: 0.208–0.440), medium‐sized individuals have a mean of 0.48 (Im) (95% CI: 0.269–0.846), and large‐sized individuals show the highest frequency at 0.71 (Im) (95% CI: 0.575–0.877). A negative binomial regression with post hoc pairwise comparisons and Tukey adjustment demonstrates a significantly higher rate of bone inflammation in large‐sized individuals compared to small‐sized individuals (*p*‐value = 0.0003).

In contrast, degenerative joint disorders (J) do not show significant differences in prevalence across size categories. The estimated frequency of degenerative joint disorders is 0.27 (J) (95% CI: 0.152–0.475) in small‐sized individuals, 0.07 (J) (95% CI: 0.021–0.218) in medium‐sized individuals, and 0.26 (J) (95% CI: 0.182–0.375) in large‐sized individuals. Although no statistically significant differences are detected, medium‐sized individuals exhibit marginally lower frequencies of degenerative joint disorders compared to both small‐ and large‐sized individuals (small vs. medium: *p*‐value = 0.095; large vs medium: *p*‐value = 0.079).

#### 3.4.2 Ontogeny

**FIGURE 8:**
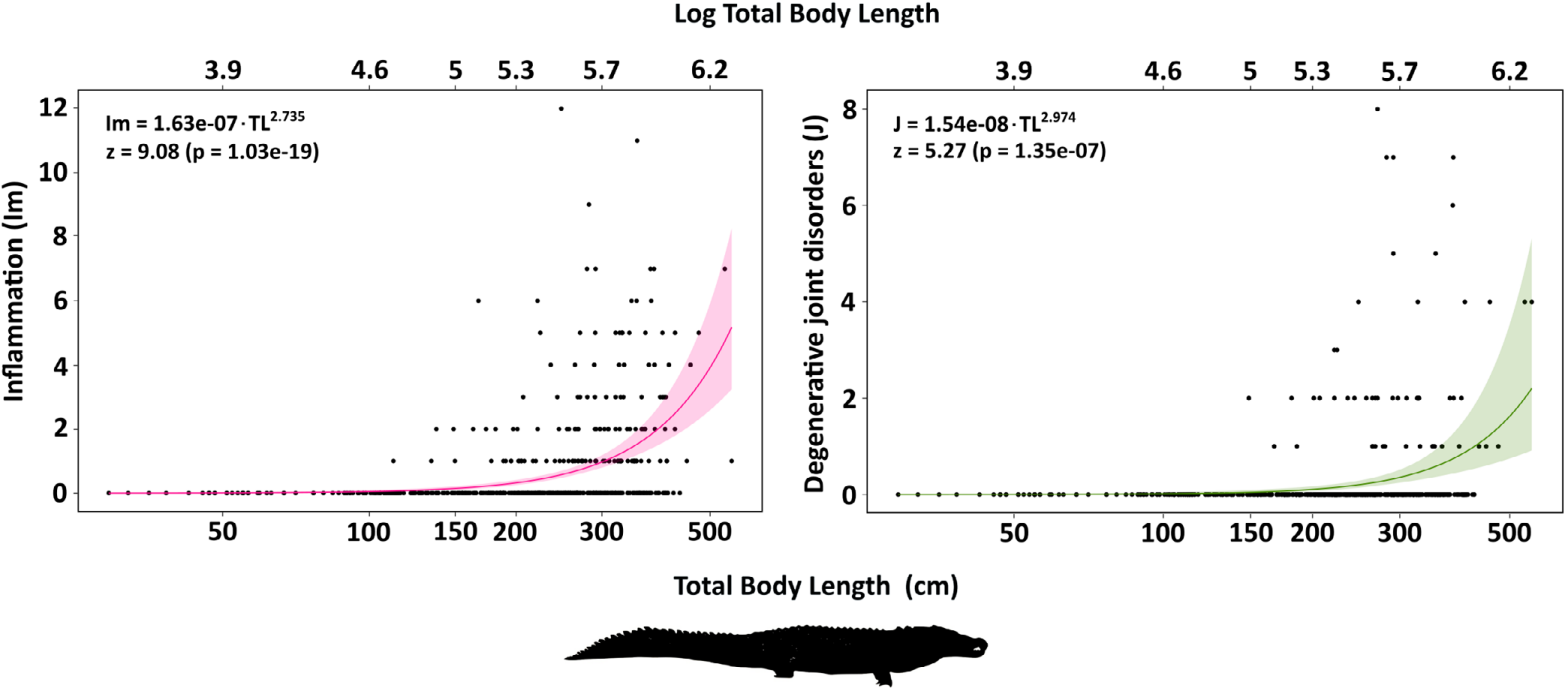
Bone inflammation and degenerative joint disorder counts against the total body length in large‐sized extant crocodilians, with regression lines, equations, and 95% confidence intervals (shaded areas), *Z* statistics, and corresponding *p*‐values.

Small‐ and medium‐sized individuals have a highly zero‐inflated dataset for bone inflammation and degenerative joint disorders, which render unfit the production of a negative binomial plot with a regression line and confidence interval against Log(TL). On the contrary, the sample of large‐sized individuals shows a significant increase in bone inflammation (*z* = 9.083 and *p*‐value = 1.05e‐19) and degenerative joint disorders (*z* = 5.272 and *p*‐value = 1.35e‐07) with Log(TL) (Figure 8).

## 4 DISCUSSION

### 4.1 Bone trauma in crocodilians

#### 4.1.1 General

Trauma represents the most common singular osteopathology identified in the crocodilian skeleton in our sample (43.9% of all pathologies). In many animals, the primary source of injury is agonistic behavior between conspecific males, particularly related to territoriality and mating. In their study on facial bite marks in tyrannosaurids, Brown et al. (2022) provide an extensive list of references and a compilation of data about the relation between agonistic behavior and trauma in extant vertebrates.

Crocodilians, among the most social of all reptilian taxa, can demonstrate a degree of conspecific tolerance during feeding, seasonal aggregation, and basking periods (Baker et al., 2022; Brien, Lang, et al., 2013; Brien, Webb, et al., 2013; Garrick & Lang, 1977; Grigg & Kirshner, 2015; Lang, 1987; Staniewicz et al., 2018). Nonetheless, close proximity during these periods, coupled with heightened aggression and territoriality during the breeding season, can provoke intraspecific conflicts, or more rarely interspecific fights (Grigg & Kirshner, 2015). Literature reports support the fact that intraspecific combat is the predominant cause of soft tissue injuries in several crocodilian species, including *C. niloticus* (Cott, 1961), *C. johnstoni* (Webb et al., 1983), *C. porosus* (Webb & Messel, 1977), *C. novaeguineae* (Montague, 1984), and *C. crocodilus* (Gorzula, 1978). Another potential source of injury is associated with feeding behavior. Crocodilians are semi‐aquatic apex predators and consume a diverse range of prey depending on their adult size, ontogenetic stage, and local prey availability (Cleuren & de Vree, 2000; Cott, 1961). Prey capture presents a moment of risk for the predator, as different prey species may possess defensive structures such as hooves, spines, flukes, horns, tusks, shells, teeth, or chemical defenses. Although the frequency of predator injury during prey capture is poorly documented, it is plausible that such encounters can cause significant trauma (Carbone et al., 2007; Mukherjee & Heithaus, 2013). Additionally, crocodilians lack the ability to restrain prey with their limbs or to chew, which likely increases the risk of injury, particularly to the skull (see Figure 7).

On the other hand, despite their status as apex predators, crocodilians may themselves fall prey to larger predators or conspecifics (Grigg & Kirshner, 2015). Failed predation attempts may result in injuries, including bone trauma. A comprehensive literature review by Somaweera et al. (2013) documented predation on crocodilians across ontogeny, involving a wide range of taxa including insects, crustaceans, fishes, amphibians, reptiles, birds, mammals, and humans. Mortality is particularly high among eggs and hatchlings, decreasing as individuals mature. Finally, cannibalistic behavior is generally opportunistic but can provoke trauma during failed attempts (Delany et al., 2011; Polis & Myers, 1985; Ugemuge et al., 2023).

The living environment can also play an important role in social and feeding behavior. Our data reveal a significant increase in bone injury frequency among captive individuals (~50%). In reptiles, most captive injuries arise from environmental factors, including enclosure design and substrate (Frye, 1981). Captive crocodilians are confined to small, often artificial environments, increasing proximity and potentially altering social behavior (Veasey et al., 1996). Indeed, captivity‐induced stress can result in both physiological and behavioral changes (DeNardo, 2006). A comparative study of captive and wild *A. mississippiensis* found that agonistic behaviors were the most frequent social interactions in captivity (Walsh et al., 2022). Therefore, zoological and wildlife facilities have increasingly sought to replicate natural conditions to reduce stress and associated trauma (Leeds et al., 2022 and references therein). The welfare of captive crocodilians is particularly important in the context of their economic value, especially in farming systems where good living conditions directly influence the product quality (Huchzermeyer, 2003; Tosun, 2013).

#### 4.1.2 Size categories and trauma

Our comparison of traumatic injury frequencies across three size categories (small, medium, and large) indicates that large‐sized crocodilians exhibit approximately twice the frequency of bone trauma as small‐sized crocodilians. These injuries are primarily observed in the four most represented taxa: *A. mississippiensis*, *C. acutus*, *C. niloticus*, and *C. porosus*. Although *C. porosus* is widely regarded as the most aggressive and territorial crocodilian species (Brien, Lang, et al., 2013; Brien, Webb, et al., 2013; Christian et al., 2013; Garrick & Lang, 1977; Webb & Messel, 1977), *A. mississippiensis* exhibited the highest mean of bone trauma among large‐sized individuals (*T* = 1.53; 95% CI: 1.0795–2.179), followed by *C. porosus* (*T* = 1.29; 95% CI: 0.9168–1.814). However, no statistically significant differences were observed among the four most represented species. In a study on agonistic behavior of crocodilians, Brien, Lang, et al. (2013) developed a scale of conspecific tolerance and aggression in juvenile crocodilians. Their findings do not quite support our results and show that most large‐sized species exhibit moderate to high levels of tolerance, except *C. porosus*, which shows low tolerance and high aggression. It is known that some large‐sized species such as *A. mississippiensis*, *C. niloticus*, and *M. niger* form large aggregations during feeding or seasonal changes, and therefore tolerate conspecifics (Grigg & Kirshner, 2015), yet they also exhibit territorial aggression, particularly in relation to food and reproduction (Cott, 1961; Grigg & Kirshner, 2015; Leeds et al., 2022). By contrast, *T. schlegelii* and *G. gangeticus*, the two large longirostrine species in our sample, are more tolerant to conspecifics (Brien, Lang, et al., 2013). Nevertheless, Maddock (2010) reported a striking behavioral interaction in two large adult male gharials (*G. gangeticus*) of approximately 4–5 m long at the Madras Crocodile Bank Trust. The two males were observed engaging in what he termed “fencing,” pushing their jaws against each other's like swords, without biting or gaping.

Caimans generally exhibit low levels of conspecific aggression. *C. latirostris* is particularly noted for low aggressive behavior and avoiding high‐risk feeding (Borteiro et al., 2009; Brien, Lang, et al., 2013; Prystupczuk et al., 2019). Some caiman species are nonetheless subject to predation by large carnivores such as jaguars and giant otters (*Pteronura brasiliensis*), which can cause trauma visible on the bone (Azevedo & Verdade, 2012; da Silveira et al., 2010; Gorzula, 1978; Perilli et al., 2016; Polisar et al., 2003; Ribas et al., 2012). Additionally, the spectacled caiman (*C. crocodilus*) can also sustain trauma from both intraspecific conflicts and aggression or predation by larger sympatric species such as *C. acutus*, with injuries most frequently affecting the tail (González‐Desales et al., 2025; Medem, 1983). Finally, boat strikes have been reported to cause traumatic injury in 36.6% of caimans in Costa Rica (Grant & Lewis, 2010).

The higher frequency of bone trauma in large‐sized individuals may reflect more intense physical interactions and increased bite force, which is more likely to inflict skeletal injuries. Bite force has been shown to increase with body size in crocodilians, regardless of phylogeny or snout morphology (Erickson et al., 2003, 2012, 2014). Furthermore, feeding behavior likely contributes to trauma frequency. Indeed, as crocodilians grow, their diet shifts toward larger prey, which increases the risks of injuries (Adame et al., 2018; Cleuren & de Vree, 2000; Cott, 1961; Drumheller et al., 2019; Grigg & Kirshner, 2015; Platt et al., 2006; Saalfeld et al., 2011). Large longirostrine crocodilians are thought to primarily consume fish and small mammals, though larger preys such as deer have occasionally been recorded in Tomistoma diet (Cleuren & de Vree, 2000; Thorbjarnarson, 1990; Grigg & Kirshner, 2015). It is important to note that agonistic behavior does not always result in physical interaction and trauma; it often serves as a communicative display involving threats prior to actual combat (Garrick & Lang, 1977; Vliet, 1989). This nuance must be considered when linking observed injuries to behavior.

#### 4.1.3 Trauma and snout shape

While snout shape clearly plays an important ecological role, our results do not reveal statistically significant differences in trauma frequency among the three snout shape categories.

Snout morphology in crocodilians is more strongly associated with ecological niche partitioning than with phylogenetic relationships, and morphospace occupation appears to be influenced predominantly by hydrodynamic efficiency and feeding biomechanics (Brochu, 2001; Busbey, 1995; Cleuren & de Vree, 2000; Drumheller & Wilberg, 2020; Pearcy, 2011; Pearcy & Wijtten, 2011; Pierce et al., 2008, 2009). Indeed, crocodilians interact with their environment primarily through their snouts, which are involved in feeding, communication, nest building, and parental care. Long and slender snouts, such as those seen in Tomistoma, Mecistops, and Gavialis, are typically adapted for capturing small prey, particularly fish. In contrast, broader snout morphotypes (brevirostrine and mesorostrine) are better suited for handling larger, more resistant prey or for crushing (Cleuren & de Vree, 2000). However, biomechanical studies have not identified substantial differences in bite force among crocodilians with different snout morphologies (Erickson et al., 2012, 2014). These findings suggest that other ecological factors, particularly those associated with body size, may exert a greater influence on bone trauma in extant crocodilians than snout morphology alone (see Sections 3.2.1 and 4.1.2).

#### 4.1.4 Trauma and ontogeny

Our data show that, within the medium‐ and large‐sized categories, bone trauma frequency increases significantly with total body length (see Figure 6). This trend is likely related to sexual maturity and an associated increase in intraspecific combat among males, as well as a dietary shift toward larger prey items as crocodilians mature (Adame et al., 2018; Cleuren & de Vree, 2000; Cott, 1961; Drumheller et al., 2019; Grigg & Kirshner, 2015; Mukherjee & Heithaus, 2013; Platt et al., 2006; Saalfeld et al., 2011). In our large‐sized category, most bone traumas occur in individuals exceeding 200 cm in total body length, which corresponds approximately to the size at which male *A. mississippiensis* reaches sexual maturity (Lance et al., 2015). Additionally, Brown et al. (2022) reviewed bite mark frequencies in 114 extant tetrapods and found that trauma frequency correlates with body size and maturity, predominantly in males. Their own dataset of 72 *A. mississippiensis* skulls similarly shows increased trauma frequency with total body length. Previous studies also support a positive correlation between injury frequency and size in crocodilians (Cott, 1961; Montague, 1984; Webb et al., 1983; Webb & Messel, 1977).

Also, we were unable to fit a model relating trauma frequency to total body length in small crocodilians, despite this group also exhibiting a normal distribution of total body length. This supports the observed lower frequency of bone trauma in small individuals compared to medium and large categories.

#### 4.1.5 Regional skeletal injury distribution

Our findings indicate that the skull is the most frequently affected skeletal region, accounting for 77.9% of all observed injuries (see Figure 7). This contrasts with previous studies on live *C. niloticus*, *C. porosus*, *C. novaeguineae*, *C. johnstoni*, and *C. crocodilus*, where the tail was the most commonly injured region, followed by the limbs, trunk, and skull (Cott, 1961; González‐Desales et al., 2025; Montague, 1984; Webb et al., 1983; Webb & Messel, 1977).

One possible explanation is the nature of injuries described in those studies, which were primarily soft tissue trauma or limb/tail amputation types that are challenging to detect in disarticulated and incomplete skeletal specimens. The deep musculature of the tail may buffer bone trauma, while superficial injuries would not be evident in skeletal material. Thus, soft tissue trauma reports may not align with the frequencies observed in osteological surveys. Additionally, bone injuries may persist much longer than soft tissue wounds due to slower turnover (Clarke, 2008; Ferreira & Duarte, 2023). Consequently, healed injuries on the skull may be overlooked in live animals but become evident postmortem. Finally, our dataset is skewed toward crania: of all the individuals with postcranial elements, only five had near‐complete skeletons (~250 postcranial bones), while 761 individuals (82% of the total number of individuals in our crocodilian sample) were represented by isolated skulls. This likely overrepresents cranial trauma in our data.

### 4.2 Non‐traumatic Osteopathologies in crocodilians

#### 4.2.1 Bone inflammation and infection

Our results indicate that bone inflammation represents the second most frequently observed pathology, accounting for 27.3% of all osteopathologies, which is concordant with numerous potential triggers. Inflammation can arise from an array of stimuli. These include infectious causes (bacteria, viruses, fungi, and parasites); physical injuries (trauma, burns, frostbite, radiation, or foreign‐body exposure); chemical irritants (corrosive agents, toxins, pollutant gases, and environmental contaminants); and immune‐driven mechanisms, including hypersensitivity reactions, autoimmune disorders, or autoinflammatory syndromes. Additionally, ischaemic damage caused by restricted blood supply, degenerative or metabolic disturbances that involve crystal deposition or lipid accumulation, or certain neoplastic processes may initiate inflammatory responses (Ryan & Majno, 1977; Schmid‐Schönbein, 2006). Bone inflammation is tightly regulated, as excessive or prolonged responses can impair healing and contribute to bone loss (Epsley et al., 2021; Lüthje et al., 2020; Medzhitov, 2008). Thus, when pathogens evade immune defenses, bone and joint infection may develop, typically either via the bloodstream (hematogenous spread) or by direct extension from a contiguous focus of infection (Frigui et al., 2024).

Infectious diseases accounted for 4.9% of the osteopathologies recorded. The semi‐aquatic environments inhabited by crocodilians are rich in potential pathogens, while their social and feeding behaviors frequently result in injuries that provide portals of entry for microbial invasion, thereby increasing the risk of inflammation and infection (Montague, 1984; Webb & Messel, 1977). Moreover, the oral and cloacal microbiota of crocodilians contain dozens of bacterial species, some potentially pathogenic, that can initiate inflammatory responses and infection when introduced into tissues via oral cavity injuries (Charruau et al., 2012; Flandry et al., 1989; Gholamhosseini et al., 2021). Nevertheless, reptiles, especially crocodilians, are generally considered to prevent infection effectively, possessing a robust immune system (Field et al., 2022; Rios & Zimmerman, 2015; Zimmerman, 2020). Most ectotherms rely heavily on innate immunity, with a comparatively less complex adaptive immune system than that of endotherms (Flajnik, 1996). The innate immune system acts rapidly as a first line of defense against a broad range of pathogens. Crocodilians in particular exhibit exceptionally potent innate immune responses, in many cases surpassing those of mammals (Conley & Shilton, 2018; Finger Jr. & Isberg, 2012; Huchzermeyer, 2003; Jangpromma et al., 2016; Kommanee et al., 2012; Merchant et al., 2003, 2005, 2006, 2014, 2024; Siroski et al., 2009; Siroski & María Soledad, 2020). Finally, reptiles, including crocodilians and birds, produce a granulomatous abscess (firm, dry, caseous mass) in an attempt to wall off an infection or foreign substances, in contrast with mammals that produce a pyogenic abscess (Huchzermeyer & Cooper, 2000). Nevertheless, a study from Ladds and Sims (1990) on young captive crocodiles (30 individuals of *C. porosus* and 22 individuals of *C. novaeguineae*) in Papua New Guinea found that 29.6% of the examined animals exhibited bacterial infections, and 31.5% were found with Coccidia (Protozoa) infection. In 13% of cases, coccidiosis was identified as the primary cause of morbidity and mortality. Also, Hamm et al. (2020) further showed that infectious diseases account for approximately 32% of reported conditions in the literature on extant birds, indicating that infection represents a non‐negligible component of archosaur diseases. Furthermore, we found no significant differences in the rate of bone infection across size categories. This contrasts with the hypotheses of Merchant et al. (2014) and Merchant et al. (2006) that larger and potentially more aggressive crocodilians possess a more efficient immune system in order to deal with more frequent injuries.

Although data on the prevalence of infectious diseases in reptiles, including crocodilians, remain scarce, our results are consistent with the widely accepted view of the crocodilian immune system's efficacy.

#### 4.2.2 Degenerative joint disorders

Degenerative joint disorders can impair mobility and thus reduce overall fitness by hindering locomotion, feeding, mating, or nesting. These pathologies account for 11.5% of osteopathological observations and were mainly observed in the postcranial axial skeleton (29.7% of non‐traumatic pathologies) and appendicular skeleton (16%). This may imply limitations in social behavior, swimming ability, or terrestrial movement of the affected individuals. Also, the feeding mechanism could be weakened as we observed a substantial amount of degenerative joint disorders in the skull region (13.9%). Notably, severe jaw joint disorders were particularly prevalent in larger and thus more mature individuals, likely resulting from overuse. Furthermore, we observed a significant increase in degenerative joint disorders with increasing total body length, and thus with age, among medium‐ and large‐sized crocodilians, consistent with established observations in mammals, particularly humans (Rychel, 2010; Shane Anderson & Loeser, 2010; Vo et al., 2013).

#### 4.2.3 Developmental and congenital anomalies

Developmental anomalies may be caused by teratogens, pollutants (e.g., pesticides, heavy metals), infections, incubation temperature fluctuations, radiation, pharmaceuticals, or genetic mutations (Finnell, 1999; Garcês et al., 2020). In our sample, congenital malformations represent one of the least frequent osteopathologies (4.4%). A recent investigation of captive crocodilians documented a substantial developmental and congenital anomalies rate of 11% in *C. acutus* embryos (Serrano et al., 2024). In contrast, other studies have reported lower frequencies; Sepúlveda et al. (2006) observed a 3% incidence of deformities in embryos and hatchlings of the American alligator (*A. mississippiensis*), while Singh and Bustard (1982) detected abnormalities in 6% of 1061 examined gharial (*G. gangeticus*) hatchlings, the majority (80%) of which involved defects of the vertebral column. Similarly low prevalence was reported by Boede and Sogbe (2000) in hatchling American (*C. acutus*) and Orinoco (*C. intermedius*) crocodiles, with fewer than 5% exhibiting malformations of the tail, forelimbs, or maxillary bones. However, these studies focused on whole‐organism malformations, while our analysis was restricted to skeletal anomalies. Thus, comparisons should be made cautiously, though our findings appear reasonably consistent. Nevertheless, we showed that the most commonly affected region was the axial skeleton (see supplementary data S2), in agreement with previous findings (Serrano et al., 2024; Singh & Bustard, 1982).

#### 4.2.4 Metabolic and nutritional bone diseases

Disorders due to metabolic and/or nutritional cause(s) (MND) were identified in only 0.6%, indicating a very low prevalence. Approximately 58% occurred in captive individuals. This supports the assumption that metabolic and nutritional bone disorders, commonly resulting from nutritional imbalances, improper husbandry practices, and/or renal dysfunction, are more prevalent in captivity (Delene et al., 2020; Huchzermeyer, 2003). Furthermore, diagnosing MND can be challenging due to the subtle nature of associated bone changes and the fragmentary, disarticulated condition of many skeletal specimens, which obscures recognition of multifocal or systemic involvement. The most frequent manifestation observed was the oblique eruption of teeth toward the external aspect of the mouth, likely caused by nutritional deficiencies and associated osteomalacia (Huchzermeyer, 1986; Jacobson, 1984).

#### 4.2.5 Neoplasia

Bone neoplasms were not identified in our study, which aligns with previous reports suggesting low cancer prevalence and hypothesized intrinsic anti‐cancer properties in crocodilians (Effron et al., 1977; Elkan & Cooper, 1976; Garner et al., 2004; Khan et al., 2019; Phosri et al., 2018; Siddiqui et al., 2017). Indeed, only a few reports of tentative bone neoplasms exist. Kälin (1936) described osteosarcoma and osteoma in two individuals of *C. crocodilus* and one individual of *C. porosus*, and Rothschild (2010) reported an osteochondroma in a *Caiman yacare* (AMNH 97300), which we examined in this study. Unfortunately, the pathological elements of AMNH 97300 cited in Rothschild (2010) as osteochondroma were absent from the collection during the investigation. Also, in a literature survey, Hamm et al. (2020) reported an overall prevalence of 1.5% for neoplasms in crocodilians and 3.1% in birds, which further supports the rarity of neoplastic diseases in extant archosaurs.

#### 4.2.6 Diseases of undetermined etiology

Osteopathologies of undetermined origin (idiopathic) represent 7.4% of findings. This modest yet non‐negligible proportion highlights the challenges of diagnosing disease from macroscopic skeletal remains alone. Bone often displays a limited range of responses to pathological stimuli, making many lesions non‐pathognomonic and thereby hindering precise etiological attribution (Assis et al., 2018; Biehler‐Gomez et al., 2020; Carotenuto et al., 2021; Wood et al., 1992).

### 4.3 Implications for the fossil record

As extant representatives of the archosaur lineage, crocodilians provide critical insights into the paleobiology and evolutionary history of extinct archosaurs, including dinosaurs (Bell & Hendrickx, 2020; Brazaitis & Watanabe, 2011; Isles, 2009; Kundanati et al., 2019). The study of crocodilian pathologies, particularly those related to feeding or intraspecific aggression (e.g., healed bite marks), offers valuable analogues for interpreting behavior in fossil archosaurs (Avilla et al., 2004; Brown et al., 2022; Butler et al., 2013). A recent study of paleopathology in abelisaurid theropods by Baiano et al. (2024) found a consistent pattern of osteopathologies: traumas were most common (55.1%), followed by undetermined pathologies (22.8%), a substantially higher rate than in our sample, likely reflecting the diagnostic limitations inherent to fossil material. Other observed conditions included bone infections (osteomyelitis, 9%), articular disorders (4.6%), congenital malformations (3%), MND (1.7%), and isolated examples of neoplasms and osteodysplasia. Nevertheless, abelisaurids were theropod dinosaurs whose life history and physiology differed from extant crocodilians. Thus, similarities or differences in osteopathological frequencies should be interpreted with caution. Nonetheless, the overall distribution of pathological conditions across these two carnivorous archosaur groups is substantially similar. While bone infection was pretty scarce in our sample, it has been described repeatedly in non‐avian dinosaurs, across all major clades (Aureliano et al., 2021, 2026; Baiano et al., 2024; Cruzado‐Caballero et al., 2023; Hamm et al., 2020; Hunt et al., 2019; and references therein). Bone neoplastic conditions were not found in our study, and reports on tentative bone neoplasm in non‐avian dinosaurs also appear rare in the literature (Baiano et al., 2024; Cruzado‐Caballero et al., 2021; Ekhtiari et al., 2020; Hamm et al., 2020 and references therein).

Overall, trauma emerges as the most common type of pathology across extant and extinct archosaurs, for skeletal and non‐skeletal lesions. The prevalence of traumatic injuries such as healed bite marks in Mesozoic archosaurs supports the hypothesis that intraspecific conflict is a plesiomorphic behavioral trait within Archosauria (Brown et al., 2022; Drumheller et al., 2020, 2023; Fiorelli, 2010; Hanna, 2002; Martin, 2013; Tanke & Currie, 1998; Wolff, 2007). Of special interest in this respect are the phytosaurs, Middle and Late Triassic archosauriforms that exhibit remarkable ecomorphological convergence with extant crocodilians. Depending on phylogenetic interpretation, they may be early diverging pseudosuchians or the sister group to Archosauria (Jones & Butler, 2018), positioning them as excellent candidates to determine the plesiomorphic situation of intraspecific behavior in archosaurs and archosauriforms, respectively. Although Abel (1922) described many bone alterations in numerous skulls that he interpreted as healed bite marks from antagonistic fights, there is currently no systematic survey of paleopathology in phytosaurs. Except for the studies by Witzmann et al. (2014) and Heckert et al. (2021), which dealt exclusively with pathological phytosaur specimens, most known phytosaur pathologies have been reported only incidentally in morphological descriptions (Case, 1930; Hungerbühler, 2000; Moodie, 1917, 1922, 1923; Stocker, 2010; von Huene, 1911). The present authors are currently working to fill this gap and compare the paleopathologies of phytosaurs with those of extant crocodilians.

## 5 CONCLUSION

This study presents the first exclusive and comprehensive assessment of osteopathologies in extant crocodilians, with broad implications for veterinary pathology, captive management, and paleobiology. Our findings confirm that trauma is the most prevalent pathological condition, consistent with trends observed in other carnivorous archosaurs, extant and extinct. We further demonstrate that bone injuries are correlated with adult body size, ontogeny, feeding strategies, and potentially species‐specific agonistic behaviors. These results offer a valuable comparative framework for future studies on both extant and extinct archosaurs. We recommend expanding this approach to other extant taxa (e.g., birds, amphibians) as well as to fossil archosauriforms to better understand the evolutionary history and functional significance of osteopathologies across the clade. In particular, comparisons with early‐diverging (basal) archosaurs could shed light on the origins and extent of behaviors associated with skeletal trauma. One promising group for such comparative analysis is the Middle to Late Triassic phytosaurs. They are represented by a large number of species, numerous specimens, and a wide geographical range, and they exhibit striking ecomorphological convergence with crocodilians. Investigating whether patterns of agonistic behavior and their paleo‐osteological correlates were already established in early archosaurs would provide critical insight into the early evolution of this major clade.

## AUTHOR CONTRIBUTIONS

**Alexis Cornille:** Conceptualization; investigation; writing – original draft; methodology; visualization; writing – review and editing; software; formal analysis; data curation; validation. **Kyla Beguesse:** Investigation; validation; writing – review and editing; data curation. **Ewan Wolff:** Data curation; validation; investigation; writing – review and editing. **Clemens Zinner:** Data curation; visualization; writing – review and editing. **Patrick Asbach:** Investigation; validation; writing – review and editing. **François Clarac:** Conceptualization; writing – review and editing; validation; supervision; methodology. **Florian Witzmann:** Conceptualization; investigation; funding acquisition; writing – review and editing; validation; methodology; project administration; supervision; resources.

## Supporting information


**Figure S1:** Supplementary Figure 1.

**Figure S2:** Supplementary Figure 2.

**Figure S3:** Supplementary Figure 3.

**Data S1:** Supporting Information.

**Data S2:** Supporting Information.
