# Supplementary material for: Skeletal pathologies in extant crocodilians as a window into the paleopathology of fossil archosaurs
Source: Anat Rec (Hoboken). 2025 Nov 15;309(10):2698–724. doi: 10.1002/ar.70079 (PMC13549245; doi:10.1002/ar.70079)
Supplement: Supplementary file 1 [file AR-309-2698-s005.docx]

**SUPPLEMENTARY DATA**

**Skeletal pathologies in extant crocodilians as a window into the paleopathology of fossil archosaurs**

**2. MATERIALS AND METHODS**

**2.1 Sample**

We examined 22,281 skeletal elements from 927 extant crocodilian individuals (Figure 1).

**2.4 Bone trauma as a proxy to behavior in extant crocodilians**

**2.4.3 Total body length**

We tested the normal distribution of total body length (TL) across the three different size categories using a Shapiro-Wilk test and density plots (Figure 2).

Shapiro-Wilk test:

**Small-sized individuals:** W = 0.9931 (p-value = 0.3955). This indicates no significant deviation from normality.

**Medium-sized individuals:** W = 0.9858 (p-value = 0.4634)**.** This indicates no significant deviation from normality.

**Large-sized individuals:** W = 0.9914 (p-value = 0.0031). This indicates a significant deviation from normality. This is likely due to subtle right skewness or kurtosis that is usually more apparent with large sample sizes (Figure 2). Nevertheless, the distribution appears close to normal visually.

**3. RESULTS**

**3.1 General crocodilian osteopathology**

Detailed results of count data for each pathological category across all taxa are available in (Table 1), and results across all sample categories are given in (Table 2).

**3.2 Bone trauma**

**3.2.1 Size Categories and trauma**

The trauma subset accounts for a relatively small proportion of the total sample (n = 287; 31.3% of 927 individuals), resulting in a high proportion of “zero” counts for trauma (T) data across all size categories: 78.2% for small-sized, 69.3% for medium-sized, and 64.9% for large-sized individuals.

**Table 1.** Osteopathologies counts by taxa.

|  | Individuals | T | Im | In | J | M | D | N | U | V |
| --- | --- | --- | --- | --- | --- | --- | --- | --- | --- | --- |
| *Taxa* |  |  |  |  |  |  |  |  |  |  |
| *A. mississippiensis* | 133 | 204 | 118 | 19 | 55 | 6 | 16 | 0 | 32 | 6 |
| *A. sinensis* | 19 | 12 | 12 | 7 | 10 | 0 | 0 | 0 | 13 | 0 |
| *C. acutus* | 59 | 69 | 59 | 18 | 32 | 1 | 1 | 0 | 12 | 1 |
| *C. crocodilus* | 109 | 38 | 16 | 2 | 14 | 0 | 5 | 0 | 4 | 0 |
| *C. intermedius* | 13 | 8 | 7 | 0 | 0 | 0 | 1 | 0 | 2 | 1 |
| *C. johnstoni* | 6 | 4 | 1 | 0 | 2 | 0 | 0 | 0 | 1 | 0 |
| *C. latirostris* | 24 | 24 | 8 | 13 | 4 | 0 | 1 | 0 | 1 | 2 |
| *C. moreletii* | 12 | 19 | 3 | 0 | 1 | 0 | 0 | 0 | 2 | 1 |
| *C. niloticus* | 113 | 128 | 75 | 12 | 20 | 0 | 13 | 0 | 23 | 4 |
| *C. novaeguineae* | 19 | 18 | 20 | 0 | 1 | 0 | 1 | 0 | 2 | 0 |
| *C. palustris* | 31 | 10 | 6 | 2 | 1 | 0 | 4 | 0 | 4 | 0 |
| *C. porosus* | 145 | 187 | 93 | 11 | 29 | 3 | 16 | 0 | 26 | 2 |
| *C. porosus X siamensis* | 2 | 1 | 5 | 0 | 4 | 0 | 0 | 0 | 0 | 0 |
| *C. rhombifer* | 6 | 2 | 0 | 0 | 0 | 0 | 0 | 0 | 1 | 0 |
| *C. siamensis* | 9 | 5 | 6 | 0 | 0 | 0 | 0 | 0 | 0 | 0 |
| *C. yacare* | 27 | 33 | 16 | 5 | 25 | 1 | 1 | 0 | 9 | 1 |
| *Crocodilia* | 11 | 12 | 4 | 4 | 3 | 1 | 0 | 0 | 2 | 0 |
| *G. gangeticus* | 28 | 14 | 5 | 0 | 0 | 0 | 1 | 0 | 1 | 1 |
| *M. cataphractus* | 36 | 13 | 12 | 1 | 2 | 0 | 4 | 0 | 0 | 0 |
| *M. niger* | 33 | 21 | 20 | 2 | 2 | 0 | 7 | 0 | 2 | 0 |
| *O. tetraspis* | 25 | 8 | 14 | 1 | 9 | 0 | 4 | 0 | 1 | 0 |
| *P. palpebrosus* | 15 | 2 | 0 | 0 | 0 | 0 | 2 | 0 | 4 | 0 |
| *P. trigonatus* | 19 | 4 | 6 | 0 | 2 | 0 | 3 | 0 | 0 | 0 |
| *T. schlegelii* | 33 | 28 | 31 | 0 | 11 | 0 | 6 | 0 | 4 | 1 |
| Total | 927 | 864 | 537 | 97 | 227 | 12 | 86 | 0 | 146 | 20 |

Model diagnostics supported the use of the negative binomial model (NB) over the Poisson model due to overdispersion (dispersion ≈ 4.58; AIC = 2199 vs. 3263). A zero-inflated negative binomial (ZINB) model provided no significant improvement (AIC = 2202).

**3.2.3 Sex**

Overdispersion was confirmed (log(θ) = –1.37, p = 0.025), validating model selection. Model comparisons favoured the simpler negative binomial model over zero-inflated negative binomial model (NB (AIC) = 199.09 vs. ZINB (AIC) = 200.06), and the Vuong test supported this choice (z = 1.86, p = 0.031).

**3.2.4 Origin**

AIC comparison slightly favoured the negative binomial model (AIC = 1375.6) over the ZINB model (AIC = 1376.8).

**Table 2.** Osteopathologies counts by categories.

|  | T | Im | In | J | M | D | N | U | V |
| --- | --- | --- | --- | --- | --- | --- | --- | --- | --- |
| **Size categories** |  |  |  |  |  |  |  |  |  |
| Large | 670 | 419 | 64 | 154 | 10 | 65 | 0 | 106 | 16 |
| Medium | 61 | 42 | 1 | 6 | 0 | 5 | 0 | 6 | 1 |
| Small | 121 | 72 | 28 | 64 | 1 | 16 | 0 | 32 | 3 |
| Undetermined | 12 | 4 | 4 | 3 | 1 | 0 | 0 | 2 | 0 |
| **Snout categories** |  |  |  |  |  |  |  |  |  |
| Brevirostrine | 337 | 203 | 49 | 109 | 7 | 38 | 0 | 68 | 10 |
| Mesorostrine | 456 | 281 | 43 | 100 | 4 | 37 | 0 | 70 | 8 |
| Longirostrine | 59 | 49 | 1 | 15 | 0 | 11 | 0 | 6 | 2 |
| Undetermined | 12 | 4 | 4 | 3 | 1 | 0 | 0 | 2 | 0 |
| **Origin** |  |  |  |  |  |  |  |  |  |
| Captivity | 127 | 87 | 15 | 39 | 7 | 19 | 0 | 16 | 0 |
| Wild | 412 | 290 | 49 | 122 | 2 | 49 | 0 | 66 | 12 |
| Unknown | 325 | 160 | 33 | 66 | 3 | 18 | 0 | 64 | 8 |
| **Sex** |  |  |  |  |  |  |  |  |  |
| Female | 19 | 34 | 4 | 6 | 0 | 1 | 0 | 4 | 1 |
| Male | 47 | 56 | 6 | 30 | 0 | 11 | 0 | 13 | 0 |
| Unknown | 798 | 447 | 87 | 191 | 12 | 74 | 0 | 129 | 19 |

**3.2.5 Ontogeny**

The comparison of model fit (AIC) between raw total body length and Log transformed total body length gave:

**T vs TL = 1394 (AIC)**

**T vs Log(TL) = 1382 (AIC)**

A difference of 11.2 AIC units is substantial, indicating that log(TL) provides a meaningfully better fit.

**Large-sized species:**

*Regression line equations:*

log(T) = –14.829 + 2.696 × log(TL) 
Back-transformed: T = 3.63 × 10⁻⁷ × TL²⋅⁶⁹⁶

McFadden’s pseudo-R² = 0.084 explaining 8.4% of the deviance.

**Table 3.** Estimated mean trauma, standard error, and 95% CI by taxa

|  | **Mean Trauma** | **SE** | **95% CI** |
| --- | --- | --- | --- |
| ***Taxa*** |  |  |  |
| *C. moreletii* | 1.583 | 0.9430 | 0.4930 – 5.085 |
| *A. mississippiensis* | 1.534 | 0.2750 | 1.0795 – 2.179 |
| *C. porosus* | 1.290 | 0.2250 | 0.9168 – 1.814 |
| *C. yacare* | 1.222 | 0.4960 | 0.5521 – 2.706 |
| *C. acutus* | 1.169 | 0.3220 | 0.6816 – 2.007 |
| *C. niloticus* | 1.133 | 0.2260 | 0.7659 – 1.675 |
| *Crocodilia* | 1.091 | 0.7010 | 0.3098 – 3.842 |
| *C. latirostris* | 1.000 | 0.4390 | 0.4231 – 2.363 |
| *C. novaeguineae* | 0.947 | 0.4700 | 0.3583 – 2.505 |
| *T. schlegelii* | 0.848 | 0.3240 | 0.4018 – 1.792 |
| *C. johnstoni* | 0.667 | 0.6160 | 0.1090 – 4.077 |
| *M. niger* | 0.636 | 0.2520 | 0.2925 – 1.385 |
| *A. sinensis* | 0.632 | 0.3310 | 0.2264 – 1.762 |
| *C. intermedius* | 0.615 | 0.3910 | 0.1772 – 2.137 |
| *C. siamensis* | 0.556 | 0.4310 | 0.1214 – 2.543 |
| *G. gangeticus* | 0.500 | 0.2240 | 0.2078 – 1.203 |
| *C. crocodilus* | 0.349 | 0.0851 | 0.2161 – 0.562 |
| *M. cataphractus* | 0.361 | 0.1520 | 0.1581 – 0.825 |
| *C. rhombifer* | 0.333 | 0.3500 | 0.0425 – 2.613 |
| *C. palustris* | 0.323 | 0.1500 | 0.1295 – 0.804 |
| *O. tetraspis* | 0.320 | 0.1660 | 0.1156 – 0.886 |
| *P. trigonatus* | 0.211 | 0.1400 | 0.0573 – 0.773 |
| *P. palpebrosus* | 0.133 | 0.1150 | 0.0247 – 0.721 |

**Medium-sized species:**

*Regression line equations:*

log(T) = –15.984 + 2.892 × log(TL) 
Back-transformed: T = 1.13 × 10⁻⁷ × TL²⋅⁸⁹²

McFadden’s pseudo-R² = 0.077 explaining approximately 7.7% of the deviance.

**Small-sized species**:

Model fitting was hindered by zero-inflation (79% of individuals had T = 0). A zero-inflated Poisson model confirmed the absence of a significant relationship between trauma and TL (β = 0.21, p-value = 0.502).

**3.2.7 Estimated mean trauma by taxon**

Detailed results of estimated bone trauma count across all crocodilian taxa are given in (Table3).

**Table 4**. General osteopathology count for each pathological category by anatomical region

|  | **T** | **Im** | **In** | **J** | **M** | **D** | **U** |
| --- | --- | --- | --- | --- | --- | --- | --- |
| **Thoracic ribs** | 13 | 12 | 7 | 20 | 0 | 2 | 0 |
| **Thoracic vertebrae** | 6 | 38 | 37 | 35 | 0 | 2 | 0 |
| **Caudal vertebrae** | 22 | 20 | 36 | 20 | 0 | 3 | 2 |
| **Cervical ribs** | 3 | 2 | 9 | 4 | 0 | 0 | 0 |
| **Cervical vertebrae** | 11 | 21 | 5 | 21 | 0 | 0 | 0 |
| **Hemal arches** | 11 | 5 | 30 | 1 | 0 | 1 | 0 |
| **Cranium** | 304 | 152 | 35 | 61 | 2 | 30 | 28 |
| **Dentition** | 54 | 7 | 4 | 0 | 5 | 3 | 16 |
| **Femora** | 6 | 5 | 8 | 2 | 0 | 0 | 0 |
| **Radii / Ulnae** | 12 | 37 | 21 | 28 | 0 | 3 | 8 |
| **Gastralia** | 13 | 0 | 0 | 0 | 0 | 0 | 0 |
| **Humeri** | 11 | 11 | 12 | 6 | 0 | 0 | 7 |
| **Interclavicle** | 1 |  | 1 | 0 | 0 | 0 | 0 |
| **Tibiae / Fibulae** | 14 | 13 | 15 | 5 | 0 | 1 | 4 |
| **Mandibles** | 198 | 155 | 23 | 45 | 5 | 20 | 16 |
| **Manus & Pes** | 40 | 41 | 119 | 18 | 0 | 8 | 1 |
| **Oral cavity** | 124 | 68 | 26 | 0 | 1 | 6 | 54 |
| **Osteoderms** | 19 | 0 | 0 | 0 | 0 | 0 | 0 |
| **Pectoral girdle** | 7 | 7 | 8 | 2 | 0 | 0 | 3 |
| **Pelvic girdle** | 2 | 7 | 15 | 8 | 0 | 2 | 7 |
| **Sacral vertebrae** | 2 | 7 | 2 | 5 | 0 | 8 | 1 |
| **Grand Total** | **873** | **608** | **413** | **281** | **13** | **89** | **147** |

**3.3 Non-traumatic osteopathologies**

**3.3.1 Skeletal distribution of non-traumatic osteopathologies**

Detailed results of osteopathology data count across anatomical skeletal regions are given in (table 4).

**3.4 Bone inflammation and degenerative joint disorders**

**3.4.2 Ontogeny**

**Joint Disorders (J) and total body length (Large-sized species):**

*Regression line equations:*

log(J) = –17.994 + 2.974 × log(TL) 
Back-transformed: J = e⁻¹⁷⋅⁹⁹⁴ × TL²⋅⁹⁷⁴

McFadden R² = 0.064 explaining approximately 6.4% of the deviance.

**Inflammation (Im) and total body length (Large-sized species):**

*Regression line equations:*

log(Im) = –15.634 + 2.735 × log(TL) 
Back-transformed: Im = 3.3 × 10⁻⁷ × TL²⋅⁷³⁵

McFadden R² = 0.088 explaining approximately 8.8% of the deviance.

**4. Discussion**

**4.2.6 Diseases of undetermined etiology**

Examples of bone pathologies with undetermined etiology are given in (Figure 3).

**FIGURES LEGENDS:**

**Figure 1.** Count of skeletal elements examined.

**Figure 2.** Density plot of total body length in large, medium and small-sized crocodilians categories, with a fitted normal distribution curve (blue line).

**Figure 3.** (A) (AMNH 31563; *A. mississippiensis*) right quadrate articular surface with dorso-ventral groove and sub-circular depression with no sign of reactive bone (white dashed rectangle); (B) (SMNS 6697; *O. tetraspis*) phalanx with reactive bone proliferation covered by soft tissue preventing a clear interpretation (white arrow); (C) (SMF 40183; *C. acutus*) palatine bones ventral aspect with well emarginated, highly vascularized, thick reactive bone proliferation; (D) (USNM 52972; *T. schlegelii*) sacral vertebra neural arch with circular full disruption (hole) of the bone surface and marginal lipping with “spiculated” bone proliferation (white dashed circle); (E) (MNHN 1919-113; *A. mississippiensis*) humerus shaft showing slight expansion (white dashed ellipse).
